# Dynalign II: common secondary structure prediction for RNA homologs with domain insertions

**DOI:** 10.1093/nar/gku1172

**Published:** 2014-11-21

**Authors:** Yinghan Fu, Gaurav Sharma, David H. Mathews

**Affiliations:** 1 Department of Biochemistry and Biophysics, University of Rochester Medical Center, 601 Elmwood Avenue, Box 712, Rochester, NY 14642, USA; 2 Center for RNA Biology, University of Rochester Medical Center, 601 Elmwood Avenue, Box 712, Rochester, NY 14642, USA; 3 Department of Electrical and Computer Engineering, University of Rochester, Hopeman 204, RC Box 270126, Rochester, NY 14627, USA; 4 Department of Biostatistics and Computational Biology, University of Rochester Medical Center, 601 Elmwood Avenue, Box 630, Rochester, NY 14642, USA

## Abstract

Homologous non-coding RNAs frequently exhibit domain insertions, where a branch of secondary structure is inserted in a sequence with respect to its homologs. Dynamic programming algorithms for common secondary structure prediction of multiple RNA homologs, however, do not account for these domain insertions. This paper introduces a novel dynamic programming algorithm methodology that explicitly accounts for the possibility of inserted domains when predicting common RNA secondary structures. The algorithm is implemented as Dynalign II, an update to the Dynalign software package for predicting the common secondary structure of two RNA homologs. This update is accomplished with negligible increase in computational cost. Benchmarks on ncRNA families with domain insertions validate the method. Over base pairs occurring in inserted domains, Dynalign II improves accuracy over Dynalign, attaining 80.8% sensitivity (compared with 14.4% for Dynalign) and 91.4% positive predictive value (PPV) for tRNA; 66.5% sensitivity (compared with 38.9% for Dynalign) and 57.0% PPV for RNase P RNA; and 50.1% sensitivity (compared with 24.3% for Dynalign) and 58.5% PPV for SRP RNA. Compared with Dynalign, Dynalign II also exhibits statistically significant improvements in overall sensitivity and PPV. Dynalign II is available as a component of RNAstructure, which can be downloaded from http://rna.urmc.rochester.edu/RNAstructure.html.

## INTRODUCTION

In the past three decades, RNA has been studied not just for its role in protein synthesis, but also for its large number of non-coding roles, where RNA directly controls cellular function (1–6). Because of the biological significance of non-coding RNAs (ncRNAs), the prediction of RNA secondary structure, i.e. the set of canonical base pairs, is now a commonly employed tool for understanding the mechanism of RNA function. Available approaches are categorized and summarized in a number of reviews (7–9).

The most accurate approach for modeling secondary structure is comparative analysis, by which the conserved structure is inferred using multiple homologs. To date, there is no approach that fully automates comparative analysis. One barrier that prevented automation is the fact that folding domains can often be inserted in one homolog relative to another. An inserted domain is a subsequence inserted in one homolog relative to one or more homologs that forms a substructure with base pairing between nucleotides that are within the inserted subsequence. For example, 9.2% of the base pairs in 60 pairs of sequences drawn from a bacterial type A RNase P RNA alignment (10) are in inserted domains. Other barriers include variation of helix and loop length and base pair opening caused by nucleotide mutations between homologous sequences.

This paper describes a novel technique that allows and accounts for domain insertions in prediction of conserved structures for two unaligned sequences. The technique was developed and demonstrated with Dynalign II, an update of Dynalign (11–14), although the principles apply generally to dynamic programming approaches for conserved structure prediction (15–21), including free energy minimization algorithms, partition function algorithms or stochastic context-free grammars. Dynalign is a pairwise RNA secondary structure prediction program that implements the Sankoff algorithm (22) for predicting the conserved structure for two unaligned homologous sequences; it has also been extended to multiple sequences with the Multilign algorithm (23) and to simultaneous structure prediction with three sequences (24). The dynamic programming recursions were updated in Dynalign II to account for the Δ*G*° in inserted domains. In addition to domain insertions, Dynalign II accommodates other types of structural variations, specifically, base pair openings and stem extensions. Base pair openings represent the situation where one of the homologs has an internal loop with nucleotides that align to base paired nucleotides in the other homolog. Stem extension represent the situation where a helix in one homolog includes a larger number of base pairs than the corresponding helix in the other homolog. The updates to Dynalign handle these structural variations with negligible increase in computational cost by using pre-computed values for the Δ*G*° for inserted domains, obtained from single sequence folding of each homolog.

The developed methodology is validated by benchmarking Dynalign II on ncRNA families that exhibit domain insertions and other structural variations, tRNA, RNase P RNA and SRP RNA. Dynalign II predicts base pairs in inserted domains with better accuracy as compared to Dynalign, and this improvement is statistically significant. Additional tests with 5S rRNA homologs provide evidence that Dynalign II encounters no degradation in performance for ncRNA families that have highly conserved secondary structure with little or no structural variation.

The following section highlights the methodology for allowing domain insertions and other aforementioned structural variations. Evaluation methods for benchmarking the algorithm and parameter selection are also discussed within the same section. Next, in the Results section, benchmarks evaluating Dynalign II for accuracy and computation time are presented. The Discussion section closes the paper with concluding remarks and a summary.

## MATERIALS AND METHODS

### Common secondary structure prediction by ΔG° minimization

Dynalign takes two sequences as input and simultaneously predicts the conserved pseudoknot-free secondary structure and the structural alignment of the sequences. A total ΔG°:

(1) \begin{equation*} \Delta G_{{\rm total}{\rm }}^o = \Delta G_1^o + \Delta G_2^o + (n_{{\rm gap}} )\Delta G_{{\rm gap\_penalty}}^o \end{equation*}

is minimized, where Δ*G*°_1_ and Δ*G*°_2_ are the folding Δ*G*°s of sequence 1 and 2, respectively, for the common structure, Δ*G*°_gap_penalty_ is the penalty per gap and *n*_gap_ is the number of gaps in the alignment between the two sequences, where the alignment is constrained to be consistent with the common structure. The Δ*G*°s are calculated according to the nearest-neighbor thermodynamic model (25–27). While these should technically be referred to as predicted Δ*G*°s, the qualifier ‘predicted’ is dropped for brevity. The original Dynalign algorithm (11–14), considers only common structures for which all base pairs in the two homologs are aligned or for which one homolog has single base pairs inserted between two aligned (conserved) base pairs. Therefore, the original Dynalign algorithm does not account for the domain insertions and other structural variations seen in RNA homologs in nature. The same observation holds true for the original Sankoff algorithm and for alternative implementations of the Sankoff algorithm (22). Dynalign II accounts for (the possibility of) domain insertions in one sequence with respect to the other by modifying the total Δ*G*° that is minimized in the process of predicting common structures to

(2) \begin{equation*} \begin{array}{*{20}l} {\Delta G_{{\rm total}}^o = \Delta G_1^o + \Delta G_2^o + (n_{{\rm gap}} )\Delta G_{{\rm gap\_penalty}}^o + } \\ {\sum\limits_i {(\Delta G_{{\rm domain\_opening}}^o + x_i \Delta G_{{\rm domain\_elongation}}^o } )} \\ \end{array} \end{equation*}

where *i* is the index of the *i*th inserted domain, *x_i_* is the length of the *i*th inserted domain and Δ*G*°_domain_opening_ and Δ*G*°_domain_elongation_ are the newly introduced Δ*G*° penalties for initiation and per nucleotide elongation of inserted domains. This is an affine model for each domain insertion into the alignment. Δ*G*°_domain_opening_ and Δ*G*°_domain_elongation_ were optimized on a training data set of known secondary structures (described later) and the value of Δ*G*°_gap_penalty_ was kept the same as in (11). The terms Δ*G*°_1_ and Δ*G*°_2_ correspond, as before, to the Δ*G*°s of the structures for sequence 1 and sequence 2 according to the nearest-neighbor thermodynamic model.

### Algorithm

Dynalign II predicts the conserved structure using a dynamic programming algorithm that generalizes the original Dynalign algorithm. In the following discussion, nucleotide positions in each sequence are indexed in 5′ to 3′ order with *i* and *j* denoting indices for sequence 1 and *k* and *l* denoting indices for sequence 2, with *i* < *j* and *k* < *l*. The optimal structure of a conserved fragment [*i*, *j*, *k*, *l*] of the two input sequences, i.e. the substructure *i* to *j* in sequence 1 aligned with the substructure *k* to *l* in sequence 2, are determined recursively from smaller to larger fragments by the dynamic programming algorithm. This determines the minimum over all possible pseudoknot-free, common secondary structures and over alignments consistent with those structures. Therefore, the algorithm guarantees the optimal structures will be found given the rules that are implemented. As with Dynalign, Dynalign II predicts structural alignments by only aligning nucleotides that are base paired in conserved base pairs. This is because the Δ*G*°_total_ in Equations (1) and (2) does not include sequence identity, so nucleotides are not aligned in loop regions.

Given two homologous RNA sequences with lengths *N*_1_ and *N*_2_, Dynalign fills two, 4D arrays of size *N*_1_ × *N*_1_ × *N*_2_ × *N*_2_. These arrays are *W*(*i*, *j*, *k*, *l*) and *V*(*i*, *j*, *k*, *l*), and they represent the Δ*G*° of putative conserved fragments of the two sequences with different conformational constraints. *V*(*i*, *j*, *k*, *l*) stores the minimum Δ*G*° of fragments [*i*, *j*, *k*, *l*], where *i* is base paired with *j*, *k* is base paired with *l* and fragment [*i*, *j*] is aligned to fragment [*k*, *l*]. *W*(*i*, *j*, *k*, *l*) stores the lowest Δ*G*° of fragments [*i*, *j*, *k*, *l*], where fragment [*i*, *j*] is aligned to fragment [*k*, *l*] and these sequence fragments represent potential branches in multibranch loops. In order to fill the arrays, auxiliary 2D arrays are needed, *W*3(*i*, *k*), *W*5(*i*, *k*), *W*1_single_(*i*, *j*), *W*2_single_(*k*, *l*), *WE*1_single_(*i*, *j*) and *WE*2_single_(*k*, *l*). *W*3(*i*, *k*) and *W*5(*i*, *k*) are fragments at the 3′ and 5′ end of the two sequences, respectively. *W*5(*i*, *k*) stores the minimum Δ*G*° of fragments [1, *i*] and [1, *k*], with no conformational constraints. *W*3(*i*, *k*) represents the minimum Δ*G*° of fragments [*i*, *N*_1_] and [*k*, *N*_2_], again with no conformational constraints. *W*1_single_(*i*, *j*), *W*2_single_(*k*, *l*), *WE*1_single_(*i*, *j*) and *WE*2_single_(*k*, *l*) are newly introduced arrays in Dynalign II for implementing domain insertions. *W*1_single_(*i*, *j*) represents the minimum Δ*G*° of fragment [*i*, *j*] of sequence 1 given nucleotides from *i* to *j* are in a branch in a multibranch loop. *W*2_single_(*k*, *l*) is analogously the minimum Δ*G*° for fragment [*k*, *l*] of sequence 2 given that nucleotides from *k* to *l* are in a branch in a multibranch loop. *WE*1_single_(*i*, *j*) represents the minimum Δ*G*° of fragment [*i*, *j*], where *i*, *j* are exterior nucleotides, i.e. there is no base pair *i’*-*j’* where *i’* < *i* < *j* < *j’*. *WE*2_single_(*k*, *l*) is for fragment [*k*, *l*], and is the analog to *WE*1_single_ for sequence 2. These four arrays are all calculated using single sequence Δ*G*° minimization routines in the RNAstructure package (28).

*V*(*i*, *j*, *k*, *l*) and *W*(*i*, *j*, *k*, *l*) are filled for both interior and exterior fragments to facilitate the prediction of suboptimal solutions (12). Interior fragments are those that span nucleotides *i* to *j* and *k* to *l*. Exterior fragments are those that span nucleotides 1 to *i*, *j* to *N*_1_, 1 to *k* and *l* to *N*_2_. For conserved structures with base pairs *i*-*j* and *k*-*l*, the lowest free energy structure possible is the sum of the *V* array for the interior and exterior fragments.

### Overview

**Figure 1.**
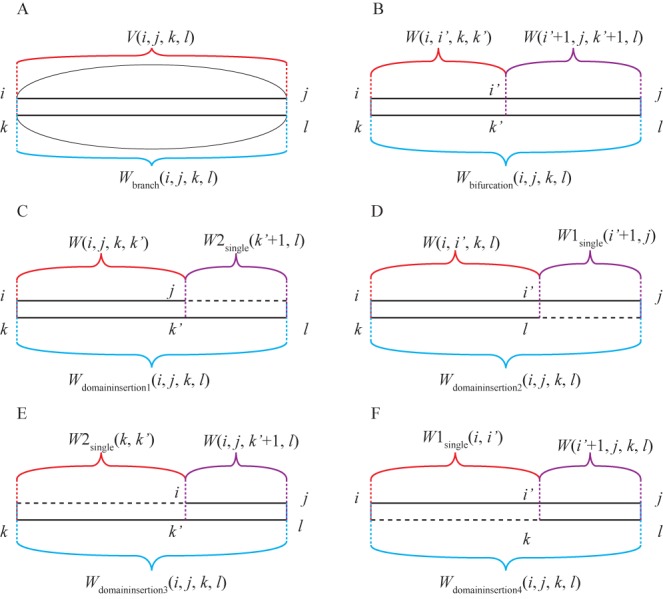
Expansion of *W*(*i*, *j*, *k*, *l*) to allow domain insertions. (A) and (B) Represent two of the original filling steps of *W*(*i*, *j*, *k*, *l*) that are for conserved domains. (C)–(F) Are expanded steps that allow consideration of inserted domains in four different positions: (C) 3′ side of sequence 2, (D) 3′ side of sequence 1, (E) 5′ side of sequence 2 and (F) 5′ side of sequence 1. The black solid lines represent sequences, black dashed lines represent gaps, black arcs represent base pairs and colored brackets are the substructures represented by the array members.

**Figure 2.**
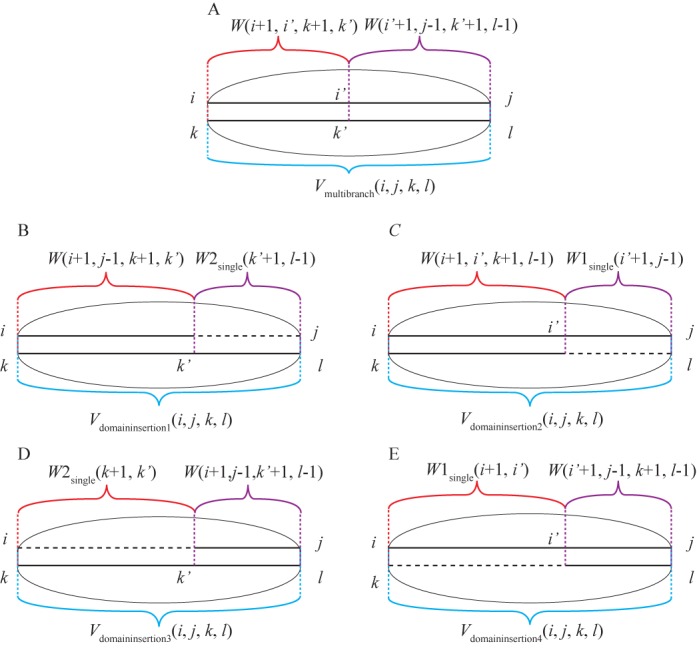
Expansion of *V*(*i*, *j*, *k*, *l*) to allows domain insertions. (A) represents the step in the original Dynalign algorithm where two conserved domains form inside a conserved base pair. (B)–(E) Illustrate how the modifications in Dynalign II account for potential inserted domains within the conserved base pair of *V*(*i*, *j*, *k*, *l*) at four positions: (B) 5′ side of sequence 2, (C) 3′ side of sequence 1, (D) 5′ side of sequence 2 and (E) the 5′ side of sequence 1.

**Figure 3.**
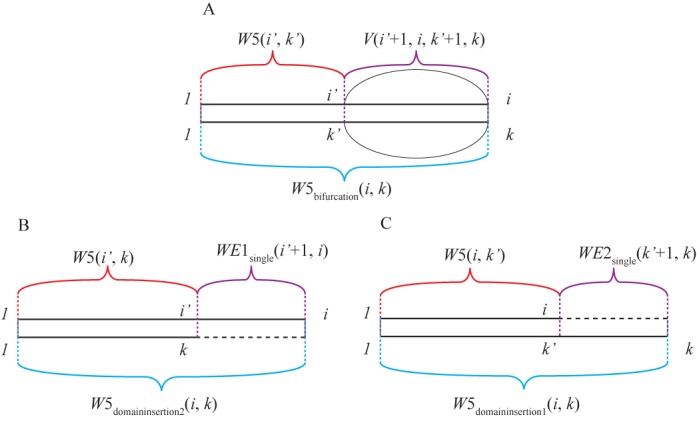
Expansion of *W*5(*i*, *k*) to account for domain insertions. (A) represents the recursion in the original Dynalign algorithm where *W*5(*i*, *k*) considers a conserved domain. (B) and (C) Represent the consideration of an inserted domain in *W*5(*i*, *k*) at two positions: (B) 3′ side of sequence 1 and (C) the 3′ side of sequence 2.

**Figure 4.**
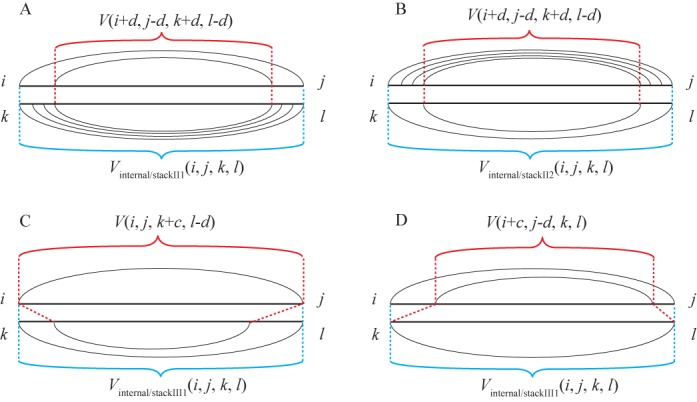
Expansion of *V*(*i*, *j*, *k*, *l*) allowing stem extension and internal loop aligning with consecutive stacking base pairs. (A) and (B) Represent an internal loop in one sequence aligned with consecutive stacking base pairs in another, where in (A) the internal loop is in sequence 1 and in (B) it is in sequence 2. (C) and (D) Represent the extension of a conserved stem, where in (C) the internal loop, stacking base pair or bulge loop is inserted in sequence 2 and in (D) it is inserted in sequence 1.

The improvements introduced by the Dynalign II algorithm are illustrated using Figures 1–4 and an abbreviated set of recursions that omit non-essential details. The full set of recursions is available in the Supplementary Materials.

To account for domain insertions, the recursions for *W*(*i*, *j*, *k*, *l*), *V*(*i*, *j*, *k*, *l*), *W*5(*i*, *k*) and *W*3(*j*, *l*) are modified from the original Dynalign algorithm. *V*(*i*, *j*, *k*, *l*) is determined as

(3) {\fontsize{8}{}{\fontsize{8}{11}\selectfont\begin{equation*} \begin{array}{*{20}l} {V(i,j,k,l) = \min [V_{{\rm hairpin}} (i,j,k,l),V_{{\rm internal/stack}} (i,j,k,l),} \\ {V_{{\rm internal/stackII}} (i,j,k,l),V_{{\rm multibranch}} (i,j,k,l) + penalty(i,j)} \\ { + penalty(k,l),V_{{\rm domain\_insertion}} (i,j,k,l) + penalty(i,j) + } \\ {penalty(k,l)]} \\ \end{array} \end{equation*}}}

where *penalty*(*i*, *j*) is the penalty term applied to A-U or G-U base pairs at the end of a helix (25,26). *V*_hairpin_(*i*, *j*, *k*, *l*) represents the Δ*G*° of hairpin loops closed by base pairs *i*-*j* and *k*-*l*. *V*_internal/stack_(*i*, *j*, *k*, *l*) represents the minimum Δ*G*° of the conserved fragment [*i*, *j*, *k*, *l*], where internal loops, bulge loops or stacking base pairs are closed by base pairs *i*-*j* and *k*-*l*. *V*_i__nternal/stackII_(*i*, *j*, *k*, *l*) accounts for new structural variations incorporated in Dynalign II that include a set of stacking base pairs aligned with an internal loop and insertion of stacking base pairs, internal loops or bulge loops of unlimited length. *V*_multibranch_(*i*, *j*, *k*, *l*) represents the minimum Δ*G*° of the conserved fragment [*i*, *j*, *k*, *l*], where multibranch loops are closed by base pairs *i*-*j* and *k*-*l*. *V*_domain_insertion_(*i*, *j*, *k*, *l*) represents the minimum Δ*G*° of the conserved fragment [*i*, *j*, *k*, *l*], where an inserted domain is formed in a loop closed by base pair *i*-*j* in sequence 1 or *k*-*l* in sequence 2. *W*(*i*, *j*, *k*, *l*) is determined as

(4) {\fontsize{8}{}{\fontsize{8}{11}\selectfont\begin{equation*} \begin{array}{*{20}l} {W(i,j,k,l) = \min [W_{{\rm extend}} (i,j,k,l),} \\ {W_{{\rm branch}} (i,j,k,l),W_{{\rm bifurcation}} (i,j,k,l),W_{{\rm domain\_insertion}} (i,j,k,l)]} \\ \end{array} \end{equation*}}}

*W*_extend_(*i*, *j*, *k*, *l*) extends substructures shorter by either one or two nucleotides in sequence 1 and/or sequence 2 with unpaired terminal nucleotides. *W*_branch_(*i*, *j*, *k*, *l*) considers the formation of a helical branch. *W*_bifurcation_(*i*, *j*, *k*, *l*) accounts for bifurcation of *W*(*i*, *j*, *k*, *l*) so that more than three helical branches can be formed in multibranch loops. *W*_domain_insertion_(*i*, *j*, *k*, *l*) represents the formation of inserted domains to *W*(*i*, *j*, *k*, *l*).

*W*5(*i*, *k*) is the minimum Δ*G*° for substructures from nucleotides 1 to *i* and from 1 to *k*. *W*3(*i*, *k*) is the minimum Δ*G*° for substructures *i* to *N*_1_ and *k* to *N*_2_, where *N*_1_ and *N*_2_ are the lengths of the sequence 1 and sequence 2, respectively:

(5) {\fontsize{8}{}{\fontsize{8}{11}\selectfont\begin{equation*} \begin{array}{l} W5(i,k){=}\min [W5(i{-}1,k){+}\Delta G^o _{{\rm gap}} ,W5(i,k{-}1){+}\Delta G^o _{{\rm gap}} , \\ W5(i - 1,k - 1),W5_{{\rm bifurcation}} (i,k),W5_{{\rm domain\_insertion}} (i,k)] \\ \end{array} \end{equation*}}}

where the first three terms account for extending shorter *W*5 fragments with unpaired nucleotides. *W*5_bifurcation_(*i*, *k*) represents the formation of conserved helical branches at the 3′ end of *W*5(*i*, *k*). *W*5_domain_insertion_(*i*, *k*) represents the formation of inserted domains at the 3′-end of *W*5(*i*, *k*). The terms for *W*3(*i*, *k*) are analogous, but involve the 3′ ends of the sequences.

Because the minimum Δ*G*° calculation for longer sequence fragments depends on the minimum Δ*G*° of shorter fragments, the array locations representing shorter fragments are filled prior to those representing longer fragments, i.e. an array location [*i’*, *j’*, *k’*, *l’*] is filled before an array location [*i*, *j*, *k*, *l*] if the fragment [*i’*, *j’*, *k’*, *l’*] is completely contained in the fragment [*i*, *j*, *k*, *l*]. After filling the arrays, the minimum Δ*G*° of the common structure is *W*5(*N*_1_, *N*_2_), which is equal to *W*3(1,1).

#### Expansion of *W*(*i*, *j*, *k*, *l*) and *V*(*i*, *j*, *k*, *l*)

In the original Dynalign algorithm, *W*(*i*, *j*, *k*, *l*) was the minimum of *W*_extend_(*i*, *j*, *k*, *l*), *W*_branch_(*i*, *j*, *k*, *l*) and *W*_bifurcation_(*i*, *j*, *k*, *l*). The last two terms are given by:

(6) \begin{equation*} \begin{array}{*{20}l} {W_{{\rm branch}} (i,j,k,l) = } \\ {V(i,j,k,l) + 2\Delta G_{{\rm helix\_terminating\_in\_MBL}}^o } \\ \end{array} \end{equation*}

(7) \begin{equation*} \begin{array}{*{20}l} {W_{{\rm bifurcation}} (i,j,k,l) = } \\ {\mathop {\min }\limits_{i {<} i' {<} j,k {<} k' {<} l} [W(i,i',k,k') + W(i' + 1,j,k' + 1,l)]} \\ \end{array} \end{equation*}

where Equation (6) represents a single, conserved branch (Figure 1A) with Δ*G*°_helix_terminating_in_MBL_ being the Δ*G*° penalty for terminating a helix of a multibranch loop and Equation (7) represents the bifurcation of the conserved domain (Figure 1B). In order to accommodate domain insertion, the calculation of *W*_domain_insertion_(*i*, *j*, *k*, *l*) is introduced in Dynalign II as:

(8) {\fontsize{8}{}{\fontsize{8}{11}\selectfont\begin{equation*} \begin{array}{*{20}l} {W_{{\rm domain\_insertion}} (i,j,k,l) = } \\ {\min [W_{{\rm domain\_insertion}1} (i,j,k,l),W_{{\rm domain\_insertion}2} (i,j,k,l),} \\ {W_{{\rm domain\_insertion}3} (i,j,k,l),W_{{\rm domain\_insertion}4} (i,j,k,l)]} \\ \end{array} \end{equation*}}}

(9) {\fontsize{8}{}{\fontsize{8}{11}\selectfont\begin{equation*} \begin{array}{*{20}l} {W_{{\rm domain\_insertion}1} (i,j,k,l) = \mathop {\min }\limits_{k {<} k' {<} l} [W(i,j,k,k') + } \\ {W2_{{\rm single}} (k' + 1,l) + \Delta G_{{\rm domain\_opening}}^o } \\ { + |l - k'|\Delta G_{{\rm domain\_elongation}}^o ]} \\ \end{array} \end{equation*}}}

(10) {\fontsize{8}{}{\fontsize{8}{11}\selectfont\begin{equation*} \begin{array}{*{20}l} {W_{{\rm domain\_insertion}2} (i,j,k,l) = } \\ {\mathop {\min }\limits_{i {<} i' {<} j} [W(i,i',k,l) + W1_{{\rm single}} (i' + 1,j) + \Delta G_{{\rm domain\_opening}}^o } \\ { + |j - i'|\Delta G_{{\rm domain\_elongation}}^o ]} \\ \end{array} \end{equation*}}}

(11) {\fontsize{8}{}{\fontsize{8}{11}\selectfont\begin{equation*} \begin{array}{*{20}l} {W_{{\rm domain\_insertion}3} (i,j,k,l) = } \\ {\mathop {\min }\limits_{i {<} i' {<} j} [W(i' + 1,j,k,l) + W1_{{\rm single}} (i,i') + \Delta G_{{\rm domain\_opening}}^o } \\ { + |i' - i + 1|\Delta G_{{\rm domain\_elongation}}^o ]} \\ \end{array} \end{equation*}}}

(12) {\fontsize{8}{}{\fontsize{8}{11}\selectfont\begin{equation*} \begin{array}{*{20}l} {W_{{\rm domain\_insertion}4} (i,j,k,l) = } \\ {\mathop {\min }\limits_{k {<} k' {<} l} [W(i,j,k' + 1,l) + W2_{{\rm single}} (k,k') + \Delta G_{{\rm domain\_opening}}^o } \\ { + |k' - k + 1|\Delta G_{{\rm domain\_elongation}}^o ]} \\ \end{array} \end{equation*}}}

where Equations (9)–(12) are illustrated by Figure 1C–F. They represent four possible positions for forming an inserted domain, the 3′ side of sequence 2 (*W*_domain_insertion1_), the 3′ side of sequence 1 (*W*_domain_insertion2_), the 5′ side of sequence 1 (*W*_domain_insertion3_) and the 5′ side of sequence 2 (*W*_domain_insertion4_). It is important to note that only one variable (*k’* or *i’*) is enumerated for each equation, and this makes the time scaling O(*N*_1_^2^ + *N*_2_^2^)for calculating *W*_domain_insertion_*(i*, *j*, *k*, *l*). This is in contrast to *W*_bifurcation_(*i*, *j*, *k*, *l*), which requires O(*N*_1_^2^*N*_2_^2^) time scaling. Therefore, the expansion to account for domain insertion in Dynalign II does not change the time complexity of Dynalign.

In *V*(*i*, *j*, *k*, *l*), *V*_multibranch_(*i*, *j*, *k*, *l*) is the minimum Δ*G*° for pairs closing multibranch loops, i.e.

(13) {\fontsize{8}{}{\fontsize{8}{11}\selectfont\begin{equation*} \begin{array}{*{20}l} {V_{{\rm multibranch}} (i,j,k,l) = \mathop {\min }\limits_{i {<} i' {<} j,k {<} k' {<} l} } \\ {[W(i + 1,i',k + 1,k') + W(i' + 1,j - 1,k' + 1,l - 1)} \\ { + 2\Delta G_{{\rm helix\_terminating\_in\_MBL}}^o + 2\Delta G_{{\rm closure\_MBL}}^o ]} \\ \end{array} \end{equation*}}}

where two conserved domains form inside base pairs *i*-*j* and *k*-*l* (Figure 2A). Δ*G*°_closure_MBL_ is the Δ*G*° penalty for the closure of a multibranch loop. With just this recursion in *V* (Equation (3)), a base pair has to close either one conserved domain (forming an internal loop/stacking base pair/bulge loop) or multiple conserved domains (forming a multibranch loop). In order to account for the situation where a conserved base pair closes a different number of branches in one homolog compared to another, the calculation of *V*_domain_insertion_(*i*, *j*, *k*, *l*) is needed:

(14) {\fontsize{8}{}{\fontsize{8}{11}\selectfont\begin{equation*} \begin{array}{*{20}l} {V_{{\rm domain\_insertion}} = } \\ {\min [V_{{\rm domain\_insertion}1} (i,j,k,l),V_{{\rm domain\_insertion}2} (i,j,k,l),} \\ {V_{{\rm domain\_insertion}3} (i,j,k,l),V_{{\rm domain\_insertion}4} (i,j,k,l)]} \\ \end{array} \end{equation*}}}

(15) {\fontsize{8}{}{\fontsize{8}{11}\selectfont\begin{equation*} \begin{array}{*{20}l} {V_{{\rm domain\_insertion}1} (i,j,k,l) = } \\ {\mathop {\min }\limits_{k {<} k' {<} l} [W(i + 1,j - 1,k + 1,k') + W2_{{\rm single}} (k' + 1,l - 1)} \\ { + 2\Delta G_{{\rm helix\_ter\min ating\_in\_MBL}}^o + 2\Delta G_{{\rm closure\_MBL}}^o + } \\ {\Delta G_{{\rm domain\_opening}}^o + |l - k' - 1|\Delta G_{{\rm domain\_elongation}}^o ]} \\ \end{array} \end{equation*}}}

(16) {\fontsize{8}{}{\fontsize{8}{11}\selectfont\begin{equation*} \begin{array}{*{20}l} {V_{{\rm domain\_insertion}2} (i,j,k,l) = } \\ {\mathop {\min }\limits_{i {<} i' {<} j} [W(i + 1,i',k + 1,l - 1) + W1_{{\rm single}} (i' + 1,j - 1)} \\ { + 2\Delta G_{{\rm helix\_terminating\_in\_MBL}}^o + 2\Delta G_{{\rm closure\_MBL}}^o + \Delta G_{{\rm domain\_opening}}^o } \\ { + |j - 1 - i'|\Delta G_{{\rm domain\_elongation}}^o ]} \\ \end{array} \end{equation*}}}

(17) {\fontsize{8}{}{\fontsize{8}{11}\selectfont\begin{equation*} \begin{array}{*{20}l} {V_{{\rm domain\_insertion}3} (i,j,k,l) = } \\ {\mathop {\min }\limits_{i {<} i' {<} j} [W(i' + 1,j - 1,k + 1,l - 1) + W1_{{\rm single}} (i + 1,i')} \\ { + 2\Delta G_{{\rm helix\_terminating\_in\_MBL}}^o + 2\Delta G_{{\rm closure\_MBL}}^o + } \\ {\Delta G_{{\rm domain\_opening}}^o + |i' - i|\Delta G_{{\rm domain\_elongation}{\rm }}^o ]} \\ \end{array} \end{equation*}}}

(18) {\fontsize{8}{}{\fontsize{8}{11}\selectfont\begin{equation*} \begin{array}{*{20}l} {V_{{\rm domain\_insertion}4} (i,j,k,l) = } \\ {\mathop {\min }\limits_{k {<} k' {<} l} [W(i + 1,j - 1,k' + 1,l - 1) + W2_{{\rm single}} (k + 1,k')} \\ { + 2\Delta G_{{\rm helix\_terminating\_in\_MBL}}^o + 2\Delta G_{{\rm closure\_MBL}}^o + } \\ {\Delta G_{{\rm domain\_opening}}^o + |k' - k|\Delta G_{{\rm domain\_elongation}{\rm }}^o ]} \\ \end{array} \end{equation*}}}

Equations (15)–(18) are illustrated in Figure 2B–E. By using *W*, *W*1 and *W*2 arrays in Equations (14)–(18), any change in the number of branching helices is accommodated because these arrays recursively consider any number of branches (see Equation (7), for example). The form of Equations (15)–(18) allow a multibranch loop in one sequence to structurally align with a single-stem loop rather than a second multibranch loop. In that case, the stem loop would be treated as a branch of a multibranch loop in terms of the energy model. This simplification in the energy model is introduced for computational efficiency.

#### Expansion of *W*3(*i*, *k*) and *W*5(*i*, *k*)

The two terms in *W*3(*i*, *k*) and *W*5(*i*, *k*) arrays exist for adding conserved branches to exterior loops:

(19) \begin{equation*} \begin{array}{*{20}l} {W5_{{\rm bifurcation}} (i,k) = } \\ {\mathop {\min }\limits_{1 \le i' {<} i,1 \le k' {<} k} [W5(i',k') + V(i' + 1,i,k' + 1,k)]} \\ \end{array} \end{equation*}

(20) \begin{equation*} \begin{array}{*{20}l} {W3_{{\rm bifurcation}} (i,k) = } \\ {\mathop {\min }\limits_{1 \le i' {<} i,1 \le k' {<} k} [W3(i',k') + V(i,i' - 1,k,k' - 1)]} \\ \end{array} \end{equation*}

where Equation (19) is demonstrated in Figure 3A.

Additional terms in the filling of *W*3(*i*, *k*) and *W*5(*i*, *k*) arrays are added to consider a domain insertion in exterior loops, *W*5_domain_insertion_(*i*, *k*) and *W*3_domain_insertion_(*i*, *k*):

(21) \begin{equation*} \begin{array}{*{20}l} {W5_{{\rm domain\_insertion}{\rm }} (i,k) = } \\ {\min [W5_{{\rm domain\_insertion}1} (i,k),W5_{{\rm domain\_insertion}2} (i,k)]} \\ \end{array} \end{equation*}

(22) \begin{equation*} \begin{array}{*{20}l} {W5_{{\rm domain\_insertion}1} (i,k) = \mathop {\min }\limits_{1 \le i' {<} i} [W5(i',k) + } \\ {WE1_{{\rm single}} (i' + 1,i) + \Delta G_{{\rm domain\_opening}}^o + } \\ {|i - i'|\Delta G_{{\rm domain\_elongation}{\rm }}^o ]} \\ \end{array} \end{equation*}

(23) \begin{equation*} \begin{array}{*{20}l} {W5_{{\rm domain\_insertion}2} (i,k) = \mathop {\min }\limits_{1 \le k' {<} k} [W5(i,k') + } \\ {WE2_{{\rm single}} (k' + 1,k) + \Delta G_{{\rm domain\_opening}}^o + } \\ {|k - k'|\Delta G_{{\rm domain\_elongation}}^o ]} \\ \end{array} \end{equation*}

(24) \begin{equation*} \begin{array}{*{20}l} {W3_{{\rm domain\_insertion}} (i,k) = } \\ {\min [W3_{{\rm domain\_insertion}1} (i,k),W3_{{\rm domain\_insertion}2} (i,k)]} \\ \end{array} \end{equation*}

(25) \begin{equation*} \begin{array}{*{20}l} {W3_{{\rm domain\_insertion}1} (i,k) = \mathop {\min }\limits_{1 \le i' {<} i} [W3(i',k) + } \\ {WE1_{{\rm single}} (i,i' - 1) + \Delta G_{{\rm domain\_opening}}^o + } \\ {|i' - i|\Delta G_{{\rm domain\_elongation}}^o ]} \\ \end{array} \end{equation*}

(26) \begin{equation*} \begin{array}{*{20}l} {W3_{{\rm domain\_insertion}2} (i,k) = \mathop {\min }\limits_{1 \le k' {<} k} [W3(i,k') + } \\ {WE2_{{\rm single}} (k,k' - 1) + \Delta G_{{\rm domain\_opening}{\rm }}^o + } \\ {|k - k'|\Delta G_{{\rm domain\_elongation}}^o ]} \\ \end{array} \end{equation*}

Equations (22) and (23) are illustrated in Figure 3B and C.

### Additional structural variations

In the original Dynalign algorithm, single base pairs could be inserted in one sequence relative to another only if they were flanked by conserved base pairs. In Dynalign II, the model is more flexible. It allows a set of stacking base pairs aligned with an internal loop and unlimited insertion of nucleotides in stacking base pairs, internal loops or bulge loops. In the original Dynalign:

(27) {\fontsize{8}{}{\fontsize{8}{11}\selectfont\begin{equation*} \begin{array}{*{20}l} {V_{{\rm internal/stack}} (i,j,k,l) = \mathop {\min }\limits_{1 \le a \le 20,1 \le b \le 20,1 \le c \le 20,1 \le d \le 20} } \\ {[V(i + a,j - b,k + c,l - d) + \Delta G_{{\rm motif}}^ \circ (i,i + a,j,j - b)} \\ { + \Delta G_{{\rm motif}}^ \circ (k,k + c,l,l - d)]} \\ \end{array} \end{equation*}}}

where Δ*G*°_motif_ (*m*, *n*, *p*, *q*) represents the Δ*G*° contributed by a motif, i.e. a base pair stack, internal loop or bulge loop closed by base pairs *m*-*p* and *n*-*q* from sequences 1 or 2. In Dynalign II, the additional types of structural alignment are realized (shown in Equations (29)-(32) and Figure 4A–D) by adding *V*_internal/stackII_(*i*, *j*, *k*, *l*):

(28) {\fontsize{8}{}{\fontsize{8}{11}\selectfont\begin{equation*} \begin{array}{*{20}l} {V_{{\rm internal/stackII}} (i,j,k,l) = } \\ {\min [V_{{\rm internal/stackII}1} (i,j,k,l),V_{{\rm internal/stackII}2} (i,j,k,l),} \\ {V_{{\rm internal/stackII}3} (i,j,k,l),V_{{\rm internal/stackII}4} (i,j,k,l)]} \\ \end{array} \end{equation*}}}

(29) {\fontsize{8}{}{\fontsize{8}{11}\selectfont\begin{equation*} \begin{array}{*{20}l} {V_{{\rm internal/stackII}1} (i,j,k,l) = \mathop {\min }\limits_{2 \le d \le 5} } \\ {[V(i + d,j - d,k + d,l - d) + \Delta G^\circ _{{\rm motif}} (i,i + d,j,j - d)} \\ { + \sum\limits_{0 \le c \le d - 1} {\Delta G^\circ _{{\rm stack}} (k + c,k + c + 1,l - c,l - c - 1)} ]} \\ \end{array} \end{equation*}}}

(30) {\fontsize{8}{}{\fontsize{8}{11}\selectfont\begin{equation*} \begin{array}{*{20}l} {V_{{\rm internal/stackII}2} (i,j,k,l) = \mathop {\min }\limits_{2 \le d \le 5} } \\ {[V(i + d,j - d,k + d,l - d) + \Delta G^\circ _{{\rm motif}} (k,k + d,l,l - d)} \\ { + \sum\limits_{0 \le c \le d - 1} {\Delta G^\circ _{{\rm stack}} (i + c,i + c + 1,j - c,j - c - 1)} ]} \\ \end{array} \end{equation*}}}

where a set of consecutive base pairs aligned with an internal loop, and Δ*G*°_stack_ (*m*, *m* + 1, *p*, *p* − 1) represents the Δ*G*° contributed by stacking base pair *m*-*p* and (*m* + 1) − (*p* − 1), which is analogous to Δ*G*°_motif_ (*m*, *m* + 1, *p*, *p* − 1).

(31) \begin{equation*} \begin{array}{*{20}l} {V_{{\rm internal/stackII}3} (i,j,k,l) = \mathop {\min }\limits_{1 \le c \le 20,1 \le d \le 20} } \\ {[V(i,j,k + c,l - d) + \Delta G^\circ _{{\rm motif}} (k,k + c,l,l - d) + } \\ {|c + d|\Delta G_{{\rm gap\_penalty}}^o ]} \\ \end{array} \end{equation*}

(32) \begin{equation*} \begin{array}{*{20}l} {V_{{\rm internal/stackII}4} (i,j,k,l) = \mathop {\min }\limits_{1 \le c \le 20,1 \le d \le 20} } \\ {[V(i + c,j - d,k,l) + \Delta G^\circ _{{\rm motif}} (i,i + c,j,j - d) + } \\ {|c + d|\Delta G_{{\rm gap\_penalty}}^o ]} \\ \end{array} \end{equation*}

where a motif *k* − *l* and (*k* + *c*) − (*l* − *d*) or *i* − *j* and (*i* + *c*) − (*j* − *d*) is inserted in sequence 2 or 1, respectively, with the gap penalty term added for each unaligned nucleotide.

### Implementation considerations and computational complexity

The full Dynalign recursions require O(*N*_1_^3^ *N*_2_^3^) time and O(*N*_1_^2^ *N*_2_^2^) memory. For typical ncRNA sequence lengths, heuristics for reducing computational time are essential in order to run on current hardware. Dynalign uses an adaptively determined banded constraint on the space of allowable nucleotide alignments. This is based on a hidden Markov model-based estimation of posterior alignment probabilities from the sequences without accounting for structure (13), which requires O(*N*_1_*N*_2_) time and memory. If the alignment constraints are approximated by a band with width *d*, i.e. aligned nucleotide indices are no further apart than (*d*/2), the algorithm reduces to O(*N*_1_^3^ *d*^3^) time and O(*N*_1_^2^ *d*^2^) memory (22). In addition to the original Dynalign, Dynalign II requires the precomputation of *W*1_single_(*i*, *j*), *W*2_single_(*k*, *l*), *WE*1_single_(*i*, *j*) and *WE*2_single_(*k*, *l*), which require O(*N*^3^) computation and O(*N*^2^) memory for each sequence. These are calculated from single sequence secondary structure predictions on each sequence, which are already performed to reduce the set of base pairs considered when filling the *V* array. This heuristic, which excludes base pairs that can only be found in relatively high Δ*G*° structures, was previously demonstrated to accelerate the calculation with no loss of accuracy (14). Thus, the time and memory complexity of Dynalign II remain the same as Dynalign, despite the additional functionality of handling a greater set of structural variations. Experimental benchmarks presented in the Results section demonstrate that, in agreement with the preceding complexity analysis, the practical time and memory requirements of Dynalign II are also almost identical to those for Dynalign.

### Evaluation

Two metrics, sensitivity and positive predictive value (PPV), were used to quantify the accuracy of structure predictions for databases of ncRNA families with known secondary structure. Sensitivity is the fraction of known base pairs that are predicted. PPV is the fraction of base pairs predicted that are in the known structure. A predicted base pair *i*-*j* is deemed correct if *i*-*j*, (*i* + *1*) − *j*, (*i* − *1*) − *j*, *i* − (*j* − *1*) or *i* − (*j* + *1*) base pair is in the known structure (13,25). This convention is adopted for two important reasons. First, base pairs in RNA structures can be dynamic, for example, single nucleotide bulges can migrate to adjacent nucleotides, as has been observed by nuclear magnetic resonance and by thermodynamic measurements (27,29–30). Second, comparative sequence analysis, which provides the ‘ground-truth’ for evaluating accuracy of secondary structure predictions, is not able to distinguish the two cases encountered when base pairs are able to migrate in position (31). For completeness, metrics computed under an exact matching requirement are also computed and reported in the Supplementary Materials. The average absolute difference of all the methods for the four families between exact and flexible matching is 0.031. The maximum difference between exact and the flexible matching is 0.05 and does not change the conclusions for the paper.

For a single sequence pair, sensitivity was calculated as the ratio of the correctly predicted to the total number of known base pairs in the structures of the two sequences, and PPV was computed as the ratio of the correctly predicted to the total number of predicted base pairs in the two sequences. Average sensitivity over an ncRNA family was calculated as the ratio of the correctly predicted to the total number of known base pairs in all the sequence pairs for the family. Average PPV over an ncRNA family was similarly computed as the ratio of the correctly predicted to the total number of predicted base pairs across all the sequence pairs for the family.

Sensitivity and PPV were also computed specifically over base pairs in inserted domains for individual ncRNA families, where complete helices and multibranch loops inserted in one sequence compared to the other homolog in the pair were identified as inserted domains. Here, sensitivity was calculated as the ratio of the correctly predicted to the total number of base pairs in the inserted domains, and PPV was computed as the ratio of the correctly predicted base pairs to the total number of base pairs in the predicted inserted domains.

Because the improvement of accuracy on individual sequence pairs can vary greatly, the one-sided paired *t*-test procedure of Xu *et al.* (32) was used to test the null hypothesis that the methods offer identical accuracy against the alternative hypothesis that Dynalign II offers higher accuracy. The one-tail *P*-value was computed to assess statistical significance of the reported improvement in accuracy.

### Dynalign II parameters

In addition to the nearest-neighbor thermodynamic parameters, Dynalign II has three additional Δ*G*° parameters: Δ*G*°_gap_penalty_, Δ*G*°_domain_opening_ and Δ*G*°_domain_elongation_. Among these, Δ*G*°_gap_penalty_ was determined by maximizing prediction accuracy on 5S rRNA sequences in the original Dynalign (11), and was found to be optimal at 0.4 kcal/mol. Δ*G*°_domain_opening_ and Δ*G*°_domain_elongation_ were determined for Dynalign II by a 2D grid search for maximizing prediction accuracy over 66 sequence pairs obtained by selecting all possible pairs from a training data set of 12 group I Intron IC1 subgroup sequences selected from a database of structures (33,34). Based on this procedure, the parameters Δ*G*°_domain_opening_ and Δ*G*°_domain_elongation_ were set to 0.5 and 0.1 kcal/mol, respectively. At these chosen values, both sensitivity and PPV were the highest over the training data set. Details of the grid search are provided in the Supplementary Materials.

## RESULTS

Structure prediction accuracy was benchmarked using four RNA families: tRNA, RNase P RNA, SRP RNA and 5S rRNA. tRNA sequences can contain variable loops that form inserted stem-loop structures. Forty tRNA sequences were randomly drawn from the Sprinzl database (35) without replacement, and all 780 sequence pairs with these sequences were chosen. The tRNA-inserted base pairs were annotated using tRNAscan-SE 1.21 (36) because the Sprinzl database does not annotate the variable loop base pairs. Base pairs in these inserted domains constitute 2.2% of all base pairs in the sequences. Note that 340 RNase P RNA sequences were randomly drawn without replacement from the bacterial type A RNA alignment on the RNase P database (10) to form 170 non-overlapping sequence pairs. Among all the base pairs in the RNase P RNA data set, 10.7% are in inserted domains. A total of 428 SRP RNA sequences were randomly drawn without replacement from the SRP database (37) to form 214 non-overlapping sequence pairs. Among all the SRP base pairs in the data set, 6.7% are in inserted domains. Twenty 5S rRNA sequences were randomly drawn from the 5S rRNA database (38) without replacement and all 190 possible sequence pairs of these sequences were considered. The 5S rRNA family has no known inserted domains and is included in the benchmark as a test for accuracy of Dynalign II on sequence pairs with little structural variation. Statistics about the pairwise sequence identities for each of the four families are provided as Supplementary Table S7. Four methods were run on the benchmark set: Dynalign II, Dynalign II without domain insertion, (original) Dynalign and Fold (a single sequence Δ*G*° minimization program from RNAstructure (28)). Dynalign II without domain insertion still included the base pair opening and stem extension functionality in order to separately test the improvement offered by each of the generalizations.

**Figure 5.**
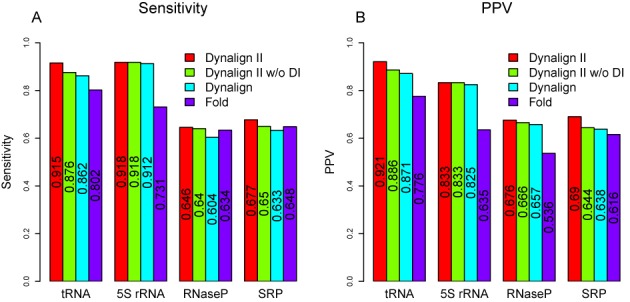
Overall structure prediction accuracy for secondary structure prediction. (A) Shows the sensitivity of the four prediction methods over homologous pairs from tRNA, 5S rRNA, RNase P RNA and SRP RNA data sets. (B) Shows the PPV of the four prediction methods on the four families. Colors represent the program used, as identified by the legends. The numerical values are indicated on the bars. The improvements in performance of Dynalign II over Dynalign and of Dynalign II over Fold are statistically significant for each RNA family Supplementary Tables S9 and S10 in the Supplementary Materials provide the *P*-values for the tests.

The overall accuracy is illustrated in Figure 5. For the RNase P RNA, SRP RNA and tRNA families, the capability to handle domain insertions and the other two structural variations each improve the sensitivity and PPV. For 5S rRNA, the capability to handle domain insertions does not improve sensitivity or PPV, which is expected given that this family does not have inserted domains. In addition, performance was stratified according to pairwise identity of sequence pairs and the results are reported in Supplementary Table S8.

**Figure 6.**
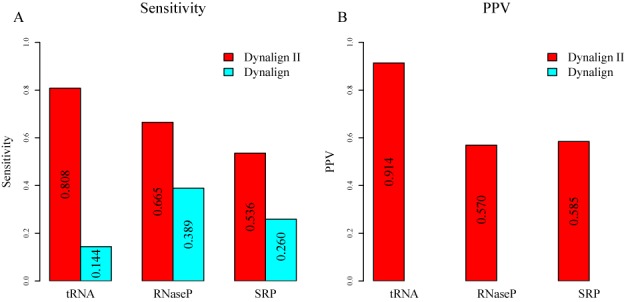
Structure prediction accuracy over base pairs in inserted domains. (A) Shows the sensitivity of Dynalign II and Dynalign on the tRNA, RNase P and SRP data sets. (B) Shows the PPV of Dynalign II and Dynalign on the tRNA, RNase P and SRP data sets. Colors represent the program used and are identified by the legends. The numerical values of the sensitivities and PPVs are indicated on the bars.

To further investigate the improvement provided by the capability to account for domain insertions, the accuracy was assessed specifically on base pairs in inserted domains. The results, shown in Figure 6, show that the sensitivity of prediction of base pairs in inserted domains is improved over the original Dynalign algorithm for the RNase P RNA, SRP RNA and tRNA families. Dynalign II also achieves a reasonable PPV in predicting base pairs in inserted domains. Note that the corresponding PPV cannot be calculated for the original Dynalign because inserted pairs are not allowed.

A one-tail paired *t*-test (32) was performed to test the statistical significance of the improvement in sensitivity and PPV for Dynalign II over Dynalign. The *P*-values computed for the test are reported in Supplementary Table S9. With the type I error rate, alpha, set to 0.05, the improvements of Dynalign II upon Dynalign are statistically significant in all cases. The statistical significance of improvements of Dynalign II upon Fold (25) were assessed using the same test and corresponding *P*-values are included in Supplementary Table S10. All the improvements are significant.

**Figure 7.**
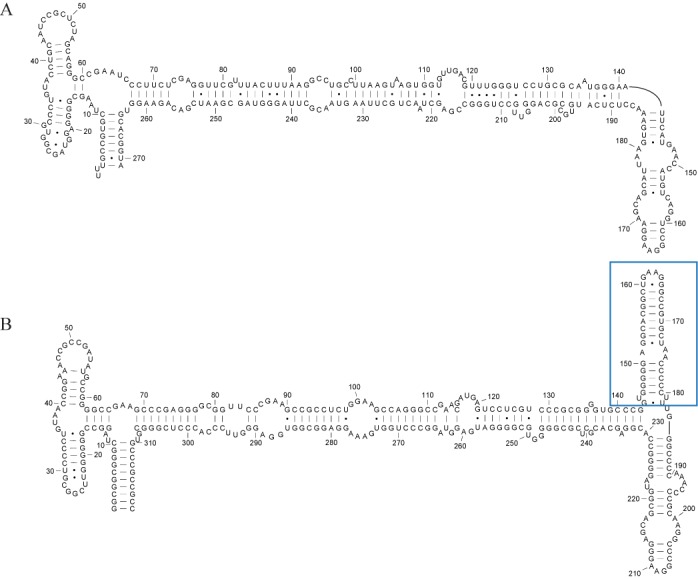
Known structures for two SRP homologs with a domain insertion in one homolog. (A) *Bacillus amyloliquefaciens* D11416 (SRP database ID: Baci.amyl._D11416) and (B) *Pyrococcus horikoshii* BA000001 (SRP database: Pyro.hori._BA000001) from the SRP database (37). The nucleotides are numbered from 5′-3′. The inserted domain in (B) is marked by a blue rectangle.

**Figure 8.**
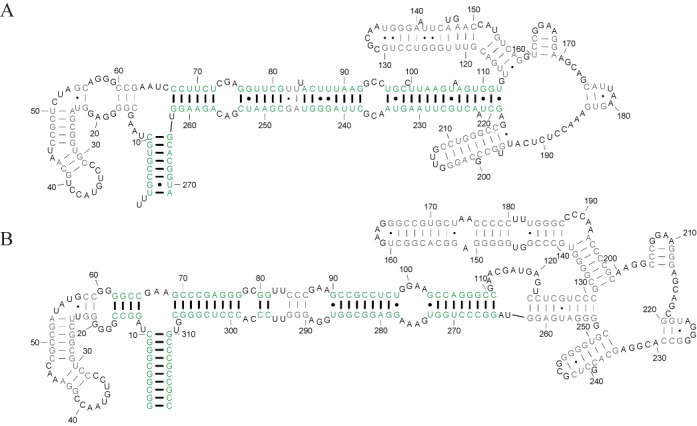
Structure predictions for the homologs in Figure 7 obtained with the original Dynalign algorithm. (A) and (B) are the Dynalign predictions for the structures of the *Bacillus amyloliquefaciens* D11416 (A) and the *Pyrococcus horikoshii* BA000001 (B), respectively. The correctly predicted base pairs are colored green and their pairs are more heavily weighted. The incorrectly predicted base pairs are colored gray and their pairs are less heavily weighted.

**Figure 9.**
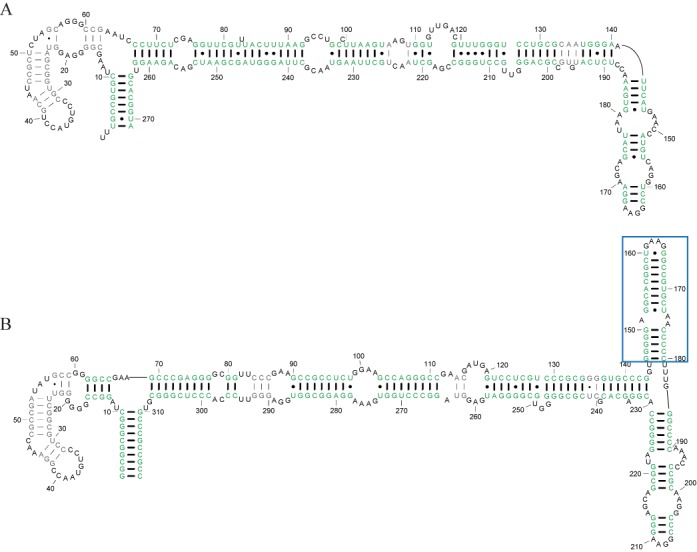
Structure prediction results for Dynalign II. (A) and (B) are the Dynalign II predictions for the structures of the *Bacillus amyloliquefaciens* D11416 and the *Pyrococcus horikoshii* BA000001, respectively. Correctly predicted base pairs are colored green and their pairs are more heavily weighted. The incorrectly predicted base pairs are colored gray and their pairs are less heavily weighted. The correctly identified inserted domain is marked by a blue rectangle.

To demonstrate the improvement provided by Dynalign II over Dynalign, an example pair of RNA homologs is illustrated in Figures 7–9. Figure 7 shows the accepted structures for two SRP RNA sequences, *Bacillus amyloliquefaciens* D11416 (SRP database ID: Baci.amyl._D11416) and *Pyrococcus horikoshii* BA000001 (SRP database ID: Pyro.hori._BA000001)(37). *horikoshii* has an inserted domain compared with *amyloliquefaciens* (indicated by a blue rectangle) in addition to the deletion and insertion of base pairs (Figure 7). The prediction made by the original Dynalign algorithm, shown in Figure 8, achieves a sensitivity of 0.55 and a PPV of 0.57. Because the original Dynalign algorithm cannot account for the domain insertion, the overall structures are incorrectly predicted. The prediction from Dynalign II, shown in Figure 9, has an improved sensitivity of 0.86 and PPV of 0.87 (Figure 9). The inserted domain is correctly identified (indicated by a blue rectangle) and the capability to account for the inserted domain also results in an overall more accurate prediction.

The results illustrate the improvement that Dynalign II offers over the original Dynalign in secondary structure prediction accuracy. Advantageously, this improvement is achieved with negligible increase of computational cost. To highlight this, the average run times and memory requirements for the original Dynalign and Dynalign II algorithms are listed in Tables 1 and 2 for RNA sequence pairs from the four families that were used in the accuracy benchmarking. The average execution times and memory requirements for Dynalign II compare favorably with those for Dynalign. For example, the average execution times for the sequence pairs from the RNase P and SRP families were 53 min:41 s and 5 h:6 min:30 s for Dynalign II, compared to 50 min:15 s and 4 h:38 min:37 s for Dynalign, on four cores of an Intel Xeon E5–2695 v2 processor. Similarly, average memory requirements for Dynalign II for sequence pairs from RNase P and SRP families was 812 MB and 1791 MB for Dynalign II, compared to 810 MB and 1790 MB for Dynalign.

**Table 1.** Average wall time required for common secondary structure prediction for 5S rRNA, tRNA, RNase P and SRP RNA homologous RNA sequence pairs

	5S rRNA	tRNA	SRP RNA	RNase P RNA
Dynalign II	55 s	18 s	5 h:6 min:30 s	53 min:41 s
Dynalign	49 s	16 s	4 h:38 min:37 s	50 min:15 s

Four cores of a 12 core Intel Xeon E5–2695 v2 processor (2.4GHz) were used for parallel computations of RNase P RNA and SRP RNA sequence pairs. One core of an Intel Xeon E5–2695 v2 processor (2.4GHz) was used for computations of 5S rRNA and tRNA sequence pairs.

**Table 2.** Average memory required for common secondary structure prediction for 5S rRNA, tRNA, RNase P and SRP homologous RNA sequence pairs

	5S rRNA	tRNA	SRP RNA	RNase P RNA
Dynalign II	76MB	57MB	1791MB	812MB
Dynalign	74MB	56MB	1790MB	810MB

## DISCUSSION

Research aimed at automating comparative sequence analysis has now been ongoing for over a decade. There is still no algorithm, however, that is as accurate at secondary structure determination as manual effort by an expert investigator (7). Two categories of obstacles prevented this. First, computational methods for comparative sequence analysis fail to properly account for structural variations among homologs. These variations include domain insertions, variations of length of helices, insertions of internal loops/bulge loops and base pair openings caused by mutation of nucleotides. Second, current computational models have only an incomplete comprehension of the factors that impact secondary structure. In particular, the influence of tertiary and pseudoknotted interactions is not included (39,40), and the thermodynamic model is imperfect.

In this paper, a novel methodology was presented to incorporate prediction of inserted domains into dynamic programming algorithms for common secondary structure prediction. The methodology was developed and implemented by updating Dynalign to Dynalign II. Figure 6 shows the dramatic impact that the proposed change has on the ability to correctly predict inserted folding domains. The improvements offered by the new technique over Dynalign in overall average prediction sensitivity and PPV are statistically significant although the numerical gains are small on average because the fraction of base pairs encountered in inserted domains in homologous structures is relatively low (Figure 5). The impact of the proposed change on specific structure predictions can be large, as shown by the example in Figures 7–9, where there is a domain insertion in one sequence relative to the other. Advantageously, the improvement in performance is achieved with negligible increase of computational cost. The new technique generalizes and enhances the overall framework provided by the Sankoff algorithm (22), and is also applicable to other comparative RNA structure analysis tools (7–9). The algorithm presented in this paper accounts for interior inserted domains in multibranch loops that terminate in one or more hairpin stem-loops. Inserted domains, however, can also be found in exterior loops of sequences with known structure, i.e. loops that contain the ends of the sequence, or they can be interior to structures, i.e. not terminating in hairpin stem loops, but terminating in conserved domains.

Another attractive area for further development is to use these improvements for conserved structure prediction for three or more homologous sequences. The work could be extended to multiple sequences, for example, by extending the Multilign method (23) to use Dynalign II instead of Dynalign. Other progressive structure alignment tools could also be adapted in similar ways.

## AVAILABILITY

Dynalign II is freely available as a component of the RNAstructure package at http://rna.urmc.rochester.edu/RNAstructure.html.

## SUPPLEMENTARY DATA

Supplementary Data are available at NAR Online.

SUPPLEMENTARY DATA
