# Supplementary material for: Dynalign II: common secondary structure prediction for RNA homologs with domain insertions
Source: Nucleic Acids Res. 2014 Nov 21;42(22):13939–48. doi: 10.1093/nar/gku1172 (PMC4267632; doi:10.1093/nar/gku1172)
Supplement: SUPPLEMENTARY DATA [file supp_42_22_13939__index.html]

Dynalign II: common secondary structure prediction for RNA homologs with domain insertions — Dynalign II: common secondary structure prediction for RNA homologs with domain insertions — SUPPLEMENTARY DATA 

# Dynalign II: common secondary structure prediction for RNA homologs with domain insertions

## SUPPLEMENTARY DATA

**Files in this Data Supplement:**

- SUPPLEMENTARY DATA
- SUPPLEMENTARY DATA
