# Supplementary material for: Dynalign II: common secondary structure prediction for RNA homologs with domain insertions
Source: Nucleic Acids Res. 2014 Nov 21;42(22):13939–48. doi: 10.1093/nar/gku1172 (PMC4267632; doi:10.1093/nar/gku1172)
Supplement: SUPPLEMENTARY DATA [file supp_gku1172_nar-02021-z-2014-File011.pdf]

Supplemental Materials for

Dynalign II: Common Secondary Structure Prediction for RNA Homologs with Domain Insertions

Yinghan Fu[1,3], Gaurav Sharma[2,3,4], David H. Mathews[1,3,4]

[1]Department of Biochemistry and Biophysics, [2]Department of Electrical and Computer Engineering, [3]Center for RNA Biology, and [4]Department of Biostatistics and Computational Biology, University of Rochester.

This document provides the Supplementary Materials for the paper (1). The first section provides the complete and detailed recursions for Dynalign II and an overview of the algorithm. The second section gives an example comparing the prediction results of Dynalign and Dynalign II on a tRNA sequence pair demonstrating the improvement brought by Dynalign II. The third section summarizes results from the grid search performed for selection of the affine gap penalty parameters for inserted domains. The fourth section provides accuracy results under an exact base pair match requirement instead of the criterion used for the results in the main manuscript. The fifth section provides sensitivity and PPV values for sequences stratified by percent identity, and the final section reports the p-values for the statistical significance of the improvements in Dynalign II over Dynalign and Dynalign II over Fold.

The Complete Recursions for Dynalign II

The dynamic programming recursions for Dynalign II operate on two four dimensional arrays: $V(i, j, k, l)$ and $W(i, j, k, l)$ and six two dimensional arrays $W3(i, k)$, $W5(i, k)$, $W1_{single}(i, j)$, $W2_{single}(k, l)$, $WE1_{single}(i, j)$, and $WE2_{single}(k, l)$ that were defined in the Methods section in the main manuscript. Among these, prior to the main Dynalign II recursions, the arrays, $W1_{single}(i, j)$, $W2_{single}(k, l)$, $WE1_{single}(i, j)$ and $WE2_{single}(k, l)$ are initialized by single sequence structure prediction algorithms, specifically the minimum ΔG° methods programmed in the RNAstructure package (2). $V(i,j,k,l)$ and $W(i,j,k,l)$ are filled for j up to $2N_1$-1 and l up to $2N_2$-1. V and W array members with index j and l bigger than N_1 and N_2 represent fragments including both the 5' and the 3' ends of the two sequences, called exterior fragments. Array members with all the indices smaller than N_1 and N_2 represent consecutive nucleotides joined by phosphodiester bonds, called interior fragments. The exterior fragment arrays are filled to facilitate the determination of suboptimal structures and energy dot plots.

Detailed Recursions for *V(i, j, k, l)*:

$$\begin{aligned}V(i,j,k,l) = \min[&V_{hairpin}(i,j,k,l), V_{multibranch}(i,j,k,l) + penalty(i,j) + penalty(k,l),\\ &V_{internal/stack}(i,j,k,l), V_{internal/stackII}(i,j,k,l),\\ &V_{domaininsertion}(i,j,k,l) + penalty(i,j) + penalty(k,l)] \quad \text{for } (j \le N1, l \le N2)\end{aligned} \quad \mathbf{, 1}$$

$$\begin{aligned}V(i,j,k,l) = \min[&V_{multibracnh}(i,j,k,l) + penalty(i,j) + penalty(k,l),\\ &V_{internal/stack}(i,j,k,l), V_{internal/stackII}(i,j,k,l), V_{domaininsertion}(i,j,k,l) + penalty(i,j) + penalty(k,l),\\ &V_{exterior}(i,j,k,l) + penalty(i,j) + penalty(k,l)] \quad \text{for } (j > N1, l > N2)\end{aligned} \quad \mathbf{, 2}$$

where $penalty(i, j)$ is the penalty term at the end of a helix that applies to AU or GU base pairs (3,4). If either the base pairs i-j or k-l are forbidden, $V(i, j, k, l)$ is set to "infinity", i.e., a large

positive value. In the above two equations, $V_{hairpin}(i,j,k,l)$ is for closing hairpin loops and $V_{exterior}(i,j,k,l)$ is for closing exterior loops. $V_{hairpin}(i,j,k,l)$ is used in interior fragments and $V_{exterior}(i,j,k,l)$ is used in exterior fragments.

$V_{hairpin}(i, j, k, l)$ considers two hairpins closed by base pairs *i-j* and *k-l*:

$$V_{hairpin}(i,j,k,l) = \Delta G^{o}{}_{hairpin}(i,j) + \Delta G^{o}{}_{hairpin}(k,l) + \Delta G^{o}{}_{gap} \mid j-i-l+k \mid, \quad \mathbf{3}$$

where $\Delta G°_{hairpin}(i, j)$ is the ΔG° of the hairpin closed by base pair *i-j*.

$V_{internal/stack}(i, j, k, l)$ considers conserved internal loops/bulge loops/helix extensions in both sequences closed by base pair *i-j* and base pair *k-l*:

$$V_{internal/stack}(i,j,k,l) = \min_{1\le a\le 20, 1\le b\le 20, 1\le c\le 20, 1\le d\le 20} [V(i+a, j-b, k+c, l-d) + \Delta G^{\circ}{}_{motif}(i, i+a, j, j-b) + \Delta G^{\circ}{}_{motif}(k, k+c, l, l-d)], \quad \mathbf{4}$$

The search over the indices *a*, *b*, *c* and *d* that determine the length of the conserved internal/bulge is constrained to an interval of 20 possibilities each to limit the overall computational complexity to $O(N^6)$, while maintaining coverage of most biologically encountered situations. $\Delta G°_{motif}(m, n, p, q)$ is the ΔG° of the motif closed by base pair *m-p* and *n-q*. When $n=m+1$ and $q=p-1$, the motif is a helix extension, meaning these two base pairs are stacking neighbors, which can be also represented as $\Delta G°_{stack}(m, n, p, q)$, when $n>m+1$ and $q<p-1$, the motif is an internal loop, and when $n>m+1$, $q=p-1$ or $n=m+1$, $q<p-1$, it is a bulge loop.

$V_{internal/stackII}(i, j, k, l)$ handles two structural variations in Dynalign II and is defined in terms of: 1) $V_{internal/stackII1}(i, j, k, l)$ and $V_{internal/stackII2}(i, j, k, l)$, which handle an internal loop aligned with a consecutive set of stacking base pairs, and 2) $V_{internal/stackII3}(i, j, k, l)$ and $V_{internal/stackII4}(i, j, k, l)$, which handle inserted stacking base pairs/internal loops/bulge loops. The recursions for these are defined as follows:

$$V_{internal/stackII}(i,j,k,l) = \min[V_{internal/stackII1}(i,j,k,l), V_{internal/stackII2}(i,j,k,l), V_{internal/stackII3}(i,j,k,l), V_{internal/stackII4}(i,j,k,l)], \quad \mathbf{5}$$

$$V_{internal/stackII1}(i,j,k,l) = \min_{2\le d\le 5}[V(i+d, j-d, k+d, l-d) + \Delta G^{\circ}{}_{motif}(i, i+d, j, j-d) + \sum_{0\le c\le d-1} \Delta G^{\circ}{}_{stack}(k+c, k+c+1, l-c, l-c-1)], \quad \mathbf{6}$$

$$V_{internal/stackII2}(i,j,k,l) = \min_{2\le d\le 5}[V(i+d, j-d, k+d, l-d) + \Delta G^{\circ}{}_{motif}(k, k+d, l, l-d) + \sum_{0\le c\le d-1} \Delta G^{\circ}{}_{stack}(i+c, i+c+1, j-c, j-c-1)], \quad \mathbf{7}$$

where a set of *c* or *d* consecutive base pairs in sequence 1 or 2, respectively, are aligned with an internal loop.

$$V_{internal/stackII3}(i,j,k,l) = \min_{1\le c\le 20, 1\le d\le 20}[V(i,j,k+c,l-d) + \Delta G^{\circ}_{motif}(k,k+c,l,l-d) + |c+d|\Delta G^{o}_{gap\_penalty}], \quad \mathbf{8}$$

$$V_{internal/stackII4}(i,j,k,l) = \min_{1\le c\le 20, 1\le d\le 20}[V(i+c,j-d,k,l) + \Delta G^{\circ}_{motif}(i,i+c,j,j-d) + |c+d|\Delta G^{o}_{gap\_penalty}], \quad \mathbf{9}$$

where the motif closed by base pair *k-l* and (*k*+*c*)-(*l-d*) or by *i-j* and (*i*+*c*)-(*j-d*) is inserted with the penalty term added.

$V_{multibranch}$(*i, j, k, l*) considers two multiple branch loops(MBL) formed in the two sequences closed by base pair *i-j* and base pair *k-l*:

$$\begin{aligned} V_{multibranch}(i,j,k,l) = &\min_{i<i'<j, k<k'<l, 1\le a\le 2, 1\le b\le 2, 1\le c\le 2, 1\le d\le 2}[W(i+a,i',k+b,k') + W(i'+1,j-c,k'+1,l-d) \\ &+ \Delta G^{o}_{dangle}(a,b,c,d) + x\Delta G^{\circ}_{unpaired\_nucleotides\_in\_MBL} + 2\Delta G^{\circ}_{MBL\_closure} \\ &+ 2\Delta G^{\circ}_{helix\_terminating\_in\_MBL} + y\Delta G^{\circ}_{gap}] \end{aligned} \quad \mathbf{, 10}$$

where *a*, *b*, *c* and *d* enumerate all the combinations of dangling ends on base pair *i-j* and *k-l*. The ΔG° sum associated with these dangling ends is $\Delta G^{\circ}_{dangle}(a, b, c, d)$, where an index of 1 indicates no dangling end and 2 indicates a dangling end. *x* is the number of unpaired nucleotides in the multibranch loop and *y* is the number of gaps, both created by the dangling ends. *i'* and *k'* are the nucleotides separating two fragments to guarantee at least two multibranch loops are formed in the two sequences.

$V_{domain\_insertion}$(*i, j, k, l*) considers two multibranch loops formed in two sequences closed by base pairs *i-j* and *k-l* with an extra domain inserted in one sequence:

$$V_{domain\_insertion}(i,j,k,l) = \min[V_{domain\_insertion1}(i,j,k,l), V_{domain\_insertion2}(i,j,k,l), V_{domain\_insertion3}(i,j,k,l), V_{domain\_insertion4}(i,j,k,l)] \quad \mathbf{.\ 11}$$

The four terms consider the four possibilities for the location of the inserted domain, viz., the 3' side of sequence 2, the 3' side of sequence 1, the 5' side of sequence 1, or the 5' side of sequence 2. Recursions for these are given as:

$$\begin{aligned} V_{domain\_insertion1}(i,j,k,l) = &\min_{k<k'<l, 1\le a\le 2, 1\le b\le 2, 1\le c\le 2, 1\le d\le 2}[W(i+a,j-c,k+b,k') + W2_{single}(k'+1,l-d) \\ &+ \Delta G^{o}_{domain\_opening} + |l-d-k'|\Delta G^{o}_{domain\_elongation} + \Delta G^{\circ}_{dangle}(a,b,c,d) + x\Delta G^{\circ}_{unpaired\_nucleotides\_in\_MBL} \\ &+ 2\Delta G^{\circ}_{MBL\_closure} + 2\Delta G^{\circ}_{helix\_terminating\_in\_MBL} + y\Delta G^{\circ}_{gap}] \end{aligned} \quad \mathbf{, 12}$$

$$V_{domain\_insertion2}(i,j,k,l)=\min_{i<i'<j,1\le a\le 2,1\le b\le 2,1\le c\le 2,1\le d\le 2}[W(i+a,i',k+b,l-d)+W1_{single}(i'+1,j-c)+\Delta G^{o}_{domain\_opening}$$
$$+|j-c-i'|\Delta G^{o}_{domain\_elongation}+\Delta G^{\circ}{}_{dangle}(a,b,c,d)+x\Delta G^{\circ}{}_{unpaired\_nucleotides\_in\_MBL}+2\Delta G^{\circ}{}_{MBL\_closure}$$
$$+2\Delta G^{\circ}{}_{helix\_terminating\_in\_MBL}+y\Delta G^{\circ}{}_{gap}]$$
, **13**

$$V_{domaini\_nsertion3}(i,j,k,l)=\min_{i<i'<j,1\le a\le 2,1\le b\le 2,1\le c\le 2,1\le d\le 2}[W(i'+1,j-c,k+b,l-d)+W1_{single}(i+a,i')+\Delta G^{o}_{domain\_opening}$$
$$+|i'-i-a+1|\Delta G^{o}_{domain\_elongation}+\Delta G^{\circ}{}_{dangle}(a,b,c,d)+x\Delta G^{\circ}{}_{unpaired\_nucleotides\_in\_MBL}+2\Delta G^{\circ}{}_{MBL\_closure}$$
$$+2\Delta G^{\circ}{}_{helix\_terminating\_in\_MBL}+y\Delta G^{\circ}{}_{gap}]$$
, **14**

$$V_{domain\_insertion4}(i,j,k,l)=\min_{k<k'<l,1\le a\le 2,1\le b\le 2,1\le c\le 2,1\le d\le 2}[W(i+a,j-c,k'+1,l-d)+W2_{single}(k+b,k')+\Delta G^{o}_{domain\_opening}$$
$$+|k'-k-b+1|\Delta G^{o}_{domain\_elongation}+\Delta G^{\circ}{}_{dangle}(a,b,c,d)+x\Delta G^{\circ}{}_{unpaired\_nucleotides\_in\_MBL}+2\Delta G^{\circ}{}_{MBL\_closure}$$
$$+2\Delta G^{\circ}{}_{helix\_terminating\_in\_MBL}+y\Delta G^{\circ}{}_{gap}]$$
, **15**

where $W1_{single}(p, q)$ is the minimum ΔG° for the fragment [*p*-*q*] in sequence 1 that will be an inserted domain inside a multibranch loop ($W2_{single}(m, n)$ is similarly defined for sequence 2). The indices *a*, *b*, *c*, *d*, *x* and *y* have the same meanings as in the calculation of $V_{multibranch}(i, j, k, l)$. The parameters $\Delta G°_{domain\_opening}$ and $\Delta G°_{domain\_elongation}$ are the initiation and elongation ΔG° parameters, respectively, for the affine gap penalty associated with inserted domains.

$V_{exterior}(i, j, k, l)$ considers the closure of exterior loops at the 5' and 3'ends of the two sequences:

$$V_{exterior}(i,j,k,l)=\min_{1\le a\le 2,1\le b\le 2,1\le c\le 2,1\le d\le 2}[W5(j-c-N_1,l-d-N_2)+W3(i+a,k+b)+\Delta G^{\circ}{}_{dangle}(a,b,c,d)]$$
, **16**

where *a*, *b*, *c* and *d* indicate dangling ends and have the same meanings as in the $V_{multibranch}(i, j, k, l)$ calculation. $W5(m, n)$ is the minimum sum of the ΔG° of fragments from the 5' end to nucleotide *m* in sequence 1 and from the 5' end to nucleotide *n* in sequence 2. $W3(p, q)$ is the minimum sum of the ΔG°of fragments from nucleotides *p* to N_1 in sequence 1and *q* to N_2 in sequence 2.

Detailed Recursions for $W(i, j, k, l)$:

$$W(i,j,k,l)=\min[W_{extend}(i,j,k,l),W_{branch}(i,j,k,l),W_{bifurcation}(i,j,k,l),W_{domain\_insertion}(i,j,k,l)]$$. **17**

The four terms over which the minimum is evaluated consider four different structural conformations and recursions for these terms follow.

***W_extend* (*i, j, k, l*) considers unpaired nucleotide addition to a smaller fragment:**

$$W_{extend}(i,j,k,l)=\min_{0\le a\le 1,0\le b\le 1,0\le c\le 1,0\le d\le 1}[W(i+a,j-b,k+c,l-d)+(|a-c|+|b-d|)\Delta G^{\circ}{}_{gap} + (a+b+c+d)\Delta G^{\circ}{}_{unpaired\_nucleotides\_in\_MBL}] \quad \text{, } \mathbf{18}$$

where a, b, c and d enumerate all the combinations of adding unpaired nucleotides at the four ends of the two fragments, where 1 indicates an added nucleotide and 0 indicates no added nucleotide. $|a-b|+|b-d|$ is the number of gaps generated, $a+b+c+d$ is the number of unpaired nucleotides generated, both caused by the deletions/insertions this step models.

***W_branch*(*i, j, k, l*) considers the formation of a helical branch:**

$$W_{branch}(i,j,k,l)=\min_{0\le a\le 1,0\le b\le 1,0\le c\le 1,0\le d\le 1}[V(i+a,j-b,k+c,l-d) + \Delta G^{\circ}{}_{dangle}(a,b,c,d)+2\Delta G^{o}_{helix\_terminating\_in\_MBL}+x\Delta G^{\circ}{}_{unpaired\_nucleotides\_in\_MBL} + y\Delta G^{\circ}{}_{gap}+penalty(i+a,j-b),penalty(k+c,l-d)] \quad \text{, } \mathbf{19}$$

where a, b, c and d enumerate all the combinations of dangling ends on base pairs $(i+a)$-$(j-b)$ and $(k+c)$-$(l-d)$. The ΔG° sum associated with these dangling ends is $\Delta G^{\circ}{}_{dangle}(a, b, c, d)$. x and y have the same meanings as in the $V_{multibranch}(i, j, k, l)$ calculation. $penalty(i+a, j-b)$ and $penalty(k+c, l-d)$ accounts for the penalty term for AU or GU pairs at the ends of helix.

***W_bifurcation*(*i, j, k, l*) considers bifurcation so that any number of branches can form in multibranch loops:**

$$W_{bifurcation}(i,j,k,l)=\min_{i<i'<j,k<k'<l}[W(i,i',k,k')+W(i'+1,j,k'+1,l)] \text{. } \mathbf{20}$$

***W_domain_insertion*(*i, j, k, l*) considers a domain inserted in one of the sequences:**

$$W_{domain\_insertion}(i,j,k,l)=\min[W_{domain\_insertion1}(i,j,k,l),W_{domain\_insertion2}(i,j,k,l), W_{domain\_insertion3}(i,j,k,l),W_{domain\_insertion4}(i,j,k,l)] \text{. } \mathbf{21}$$

These four terms consider the four possibilities identical to those considered in the computation of $V_{domain\_insertion}(i, j, k, l)$:

$$W_{domain\_insertion1}(i,j,k,l)=\min_{k<k'<l}[W(i,j,k,k')+W2_{single}(k'+1,l)+\Delta G^{o}_{domain\_opening} +|l-k'|\Delta G^{o}_{domain\_elongation}] \quad \text{, } \mathbf{22}$$

$$W_{domain\_insertion2}(i,j,k,l)=\min_{i<i'<j}[W(i,i',k,l)+W1_{single}(i'+1,j)+\Delta G^{o}_{domain\_opening} +|j-i'|\Delta G^{o}_{domain\_elongation}] \quad \text{, } \mathbf{23}$$

$$W_{domain\_insertion3}(i,j,k,l) = \min_{i<i'<j}[W(i'+1,j,k,l) + W1_{single}(i,i') + \Delta G^o_{domain\_opening} + |i'-i+1|\Delta G^o_{domain\_elongation}]$$, **24**

$$W_{domain\_insertion4}(i,j,k,l) = \min_{k<k'<l}[W(i,j,k'+1,l) + W2_{single}(k,k') + \Delta G^o_{domain\_opening} + |k'-k+1|\Delta G^o_{domain\_elongation}]$$. **25**

The rationale for these recursions is similar to that for the calculation of $V_{domain\_insertion}(i, j, k, l)$.

Detailed Recursions for *W5*(*i*,*k*):

$$W5(i,k) = \min[W5(i-1,k) + \Delta G^\circ_{gap}, W5(i,k-1) + \Delta G^\circ_{gap}, W5(i-1,k-1), W5_{bifurcation}(i,k), W5_{domain\_insertion}(i,k)]$$. **26**

W5**$_{bifurcation}$(*i*,*k*) considers helix branches forming in the two sequences:**

$$W5_{bifurcation}(i,k) = \min_{0\le i'<i, 0\le k'<k, 1\le a\le 2, 0\le b\le 1, 1\le c\le 2, 0\le d\le 1}[W5(i',k') + V(i'+a, i-b, k'+c, k-d) + \Delta G^o_{dangle}(a,b,c,d) + y\Delta G^o_{gap} + penalty(i'+a, i-b) + penalty(k'+c, k-d)]$$,
27

where *a*, *b*, *c* and *d* enumerate all the combinations of dangling ends on base pairs (*i'*+*a*)-(*i*-*b*) and (*k'*+*c*)-(*k*-*d*). The ΔG° sum associated with these dangling ends is $\Delta G^\circ_{dangle}(a, b, c, d)$. *y* is the number of gaps, created by the dangling ends.

W5**$_{domain\_insertion}$(*i*, *k*) considers an extra domain inserted in one sequence:**

$$W5_{domain\_insertion}(i,k) = \min[W5_{domain\_insertion1}(i,k), W5_{domain\_insertion2}(i,k)]$$. **28**

These two terms in the minimization consider different positions for the insertion of the extra domain. In the calculation of $W5_{domain\_insertion}(i, k)$, the extra domain can only be inserted at the 3' end of either sequence. Therefore there are only two possibilities to be considered:

$$W5_{domain\_insertion1}(i,k) = \min_{0\le i'<i}[W5(i',k) + WE1_{single}(i'+1,i) + \Delta G^o_{domain\_opening} + |i-i'|\Delta G^o_{domain\_elongation}]$$, **29**

$$W5_{domain\_insertion2}(i,k) = \min_{0\le k'<k}[W5(i,k') + WE2_{single}(k'+1,k) + \Delta G^o_{domain\_opening} + |k-k'|\Delta G^o_{domain\_elongation}]$$, **30**

where $WE1_{single}(m, n)$ is the minimum ΔG° of fragment from nucleotide *m* to *n* in sequence 1 with *m* and *n* being nucleotides in an exterior loop. $WE2_{single}(m, n)$ corresponds to the analogous fragment in sequence 2.

$W5(0, k')$ is initialized to $k'\Delta G^{o}_{gap}$.

Recursions for *W3*(*i, k*):

$W3(i, k)$ is calculated in a manner similar to $W5(i, k)$.

Dynalign II Algorithm Overview

input: 2 homologous RNA sequences s_1, s_2.
output: The alignment and structures for the 2 sequences.

```
begin
    //Pre-compute required minimum ΔG° for fragments of individual sequences
    Compute 2-D arrays W1_single and WE1_single using Single_sequence_fold(s_1);
    Compute 2-D arrays W2_single and WE2_single using Single_sequence_fold(s_2);
    /*Use dynamic programming recursions to obtain the minimum ΔG° for a common
    secondary structure and conforming alignment for the two input sequences. Account for
    domain insertions using single-sequence fold information */
    Compute 4-D arrays V and W using Dynalign_fold(s_1, s_2, W1_single, WE1_single, W2_single,
WE2_single);
    /* Traceback to identify the minimum ΔG° common secondary structure and associated
    alignment */
    Determine secondary structure and alignment using Traceback(V, W);
end

//Definitions of the functions.
function Single_sequence_fold(s_k):
    for h←0 to N_k (length of s_k) do
        for i←1 to N_k-h do
            j=i+h;
            /* Find the minimum ΔG° of the fragment i-j of s using minimum ΔG°
previously computed for smaller sub-fragments */
            Compute Wk_single(i, j) and WEk_single(i, j);
        end
    end
end

function Dynalign_fold(s_1, s_2, W1_single, WE1_single, W2_single, WE2_single):
    minloop=5;// The parameter representing the minimum size of a stem loop
    for j←minloop to N_1 do
        for i←j-1 to 1 do
            for k←N_2 to 1 do
                for l=k+minloop to N_2 do
                    /* Find the minimum ΔG° of common secondary structure
and a conforming alignment of the fragments i-j and k-j of s_1, s_2. The calculation is carried out
according to Equations 1-15 and 17-25. Domain insertions are accounted for in Equations 11-15
and 21-25. */
                    Compute W(i, j, k, l) and V(i, j, k, l);
                end
            end
        end
    end
    for i←1 to N_1 do
        for k←1 to N_2 do
            /* Find the minimum ΔG° of common secondary structure and a
conforming alignment of the fragments 1-i and 1-k of s_1,s_2. The calculation is carried out
according to Equations 26-30. Domain insertions are accounted for in Equations 28-30. */
            Compute W5(i, k);
        end
    end
```

for $i \leftarrow N_1$ **to** 1 **do**
for $k \leftarrow N_2$ **to** 1 **do**
/* Find the minimum ΔG° of common secondary structure and a conforming alignment of the fragments i-N_1 and k-N_2 of s_1, s_2. The related equations are not explicitly written, but are directly analogous to Equations **26-30** used for calculating *W5* array members. */
Compute $W3(i, k)$;
end
end
for $j \leftarrow N_1+1$ **to** $2N_1-1$ **do**
for $i \leftarrow N_1$ **to** $j-N_1+1$ **do**
for $k \leftarrow N_2$ **to** 1 **do**
for $l \leftarrow N_2+1$ **to** N_2+k **do**
/* Find the minimum ΔG° of common secondary structure and a conforming alignment of the fragments i-j and k-l of s_1, s_2 where j and l are larger than N_1 and N_2, respectively (exterior fragments). The calculation is carried out according to Equations **1-25**. Domain insertions are accounted for in Equations **11-15** and **21-25**. */
Compute $W(i, j, k, l)$ and $V(i, j, k, l)$;
end
end
end
end
end

function Traceback(V, W):
/* Determine the optimal common secondary structure and conforming alignment corresponding to the minimum ΔG° determined in Dynalign_fold(s_1, s_2, $W1_{single}$, $WE1_{single}$, $W2_{single}$, $WE2_{single}$) by recursively tracing back */
Find the indices i,j,k,l for which $V(i, j, k, l)+ V(j, i+N_1, l, k+N_2)$ is minimum. This minimum is the minimum ΔG° of the common structure of the 2 sequences. Denote the indices achieving the minimum by i', j', k', l';
Find the folding/aligning path by which $V(i', j', k', l')$ and $V(j', i'+N_1, l', k'+N_2)$ get their values according to Equations **1-30**; Identify the base pairs and the alignment in $V(i', j', k', l')$ and $V(j', i'+N1, l', k'+N2)$ according to Equations **4-9**, **19**, and **27** (5);
end

Prediction Results of Dynalign and Dynalign II on a tRNA Sequence Pair

An example is illustrated in Figures S1 and S2 to demonstrate the improvement in Dynalign II over Dynalign. Figure S1 shows the accepted structures for two tRNA sequences, *Bacillus subtilis* Ala-tRNA (Sprinzl ID: RA1540) and *Spinacia oleracea* Leu-tRNA (Sprinzl ID: RL3280) (6). RL3280 has an inserted domain compared with RA1540 (indicated by a blue rectangle) in addition to the deletion and insertion of base pairs (Figure S1). The prediction made by the original Dynalign algorithm, shown in Figure S2 A and B, have a mean sensitivity of 0.318 and PPV of 0.298. Because the original Dynalign algorithm does not account for the domain insertion, the overall topology of the structures is incorrectly predicted. Only one correct helix is identified (annotated as red in Figure S2A, B). The predictions obtained with Dynalign II are shown in Figure S2 C and D. Dynalign II significantly improves the sensitivity to 0.972 and PPV to 1.00. The inserted domain is correctly identified (indicated by a blue rectangle). This results in an overall more accurate prediction.

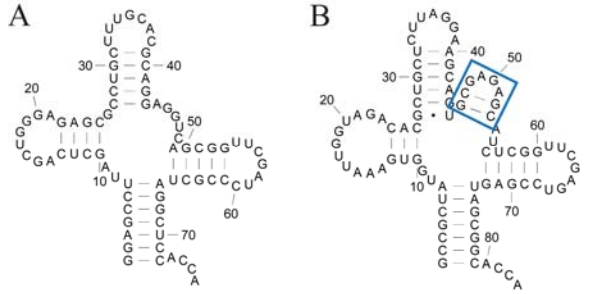

Figure S1. Structure comparison between two tRNA sequences, (A) *Bacillus subtilis* Ala-tRNA (Sprinzl ID: RA1540) and (B) *Spinacia oleracea* Leu-tRNA (Sprinzl ID: RL3280) from Sprinzl tRNA database (6). The nucleotides are numbered from 5'-3'. The inserted domain in (B) is marked by a blue rectangle.

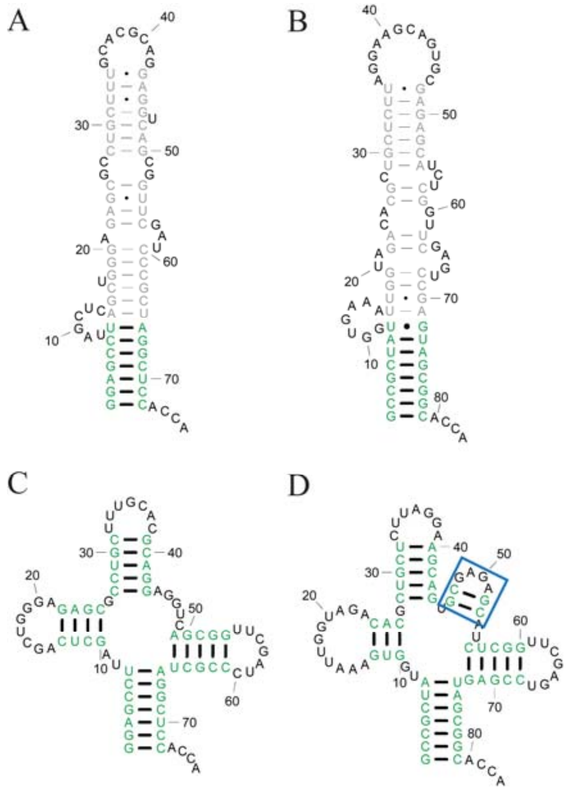

Figure S2. The structure prediction results for Dynalign and Dynalign II. In panels A and B are the Dynalign predictions for the structures of the *Bacillus subtilis* Ala-tRNA (A) and the *Spinacia oleracea* Leu-tRNA (B). In (C) and (D) are the Dynalign II predictions for the structures of the *Bacillus subtilis* Ala-tRNA (C) and the *Spinacia oleracea* Leu-tRNA (D). Correctly predicted base pairs are colored red. Correctly identified inserted domain is marked by a blue rectangle.

Grid Search for Optimal Domain Insertion Parameters $\Delta G^{o}_{domain\_opening}$ and $\Delta G^{o}_{domain\_elongation}$

A two-dimensional grid search for optimal $\Delta G^{o}_{domain\_opening}$ and $\Delta G^{o}_{domain\_elongation}$ was performed on 66 sequence pairs selected from twelve group I Intron IC1 subgroup sequences. The values for sensitivity and PPV over the grid of parameter values considered in the search are shown in the following tables.

Table S1. Sensitivity for inserted domains as a function of parameter values on the search grid. The cells in the table are colored according to their value. Backslashes in the cells indicate unmeasured data.

$\Delta G^{o}_{domain\_opening}$(kcal/mol) / $\Delta G^{o}_{domain\_elongation}$(kcal/mol)	0.2	0.5	0.8	1.5	2.5
0.05	0.532	0.532	0.534	0.535	0.536
0.075	0.556	0.552	0.552	\	\
0.1	0.58	0.578	0.575	0.573	\
0.15	0.577	0.58	0.581	\	\
0.2	0.544	0.545	0.537	0.536	0.546
0.4	\	0.535	\	0.533	0.532
0.6	\	0.53	\	0.525	0.517

Table S2. PPV for inserted domains as a function of parameter values on the search grid. The cells in the table are colored according to their value. Backslashes in the cells indicate unmeasured data.

$\Delta G^{o}_{domain\_opening}$(kcal/mol) / $\Delta G^{o}_{domain\_elongation}$(kcal/mol)	0.2	0.5	0.8	1.5	2.5
0.05	0.543	0.54	0.542	0.544	0.541
0.075	0.593	0.589	0.583	\	\
0.1	0.61	0.617	0.604	0.600	\
0.15	0.594	0.594	0.595	\	\
0.2	0.568	0.568	0.560	0.561	0.570
0.4	\	0.563	\	0.561	0.558
0.6	\	0.555	\	0.551	0.542

According to Table S1-S2, $\Delta G^{o}_{domain\_opening}$=0.5 kcal/mol and $\Delta G^{o}_{domain\_elongation}$=0.1 kcal/mol are chosen as the domain insertion parameters for Dynalign II.

Prediction Accuracy Evaluated Under an Exact Base Pair Matching Criterion

The statistics summarizing the accuracy of structure predictions provided in the main manuscript (and in the preceding section) allow one nucleotide index in base pairs to differ by +/-1 when comparing predictions with known secondary structures. This accounts for the fact that comparative analysis often cannot resolve the exact pair, and because pairs can be dynamic. Here, corresponding statistics for prediction accuracy under an exact base pair matching requirement for the comparison between predictions and known structures are provided, i.e. a predicted base pair *i-j* is deemed correct if and only if the base pair *i-j* is in the known structure.

Table S3. Overall sensitivity evaluated under an exact base pair matching criterion

	Dynalign II	Dynalign II w/o DI	Dynalign	Fold
tRNA	0.888	0.850	0.835	0.756
5S rRNA	0.906	0.906	0.885	0.709
RNase P RNA	0.616	0.608	0.574	0.604
SRP RNA	0.637	0.611	0.595	0.609

Table S4. Overall PPV evaluated under an exact base pair matching criterion

	Dynalign II	Dynalign II w/o DI	Dynalign	Fold
tRNA	0.894	0.860	0.845	0.730
5S rRNA	0.821	0.821	0.797	0.614
RNase P RNA	0.639	0.625	0.618	0.507
SRP RNA	0.643	0.600	0.594	0.572

Table S5. Structure prediction sensitivity for base pairs occurring in inserted domains evaluated under an exact base pair matching criterion.

	Dynalign II	Dynalign
tRNA	0.808	0.144
RNase P RNA	0.635	0.353
SRP RNA	0.502	0.244

Table S6. PPV for base pairs occurring in inserted domains evaluated under an exact base pair matching criterion.

	Dynalign II	Dynalign
tRNA	0.883	NA
RNase P RNA	0.547	NA
SRP RNA	0.547	NA

Table S7. Sequence identity (identical nucleotides/aligned nucleotides) statistics for tRNA, 5S rRNA, RNase P RNA and SRP RNA.

	Average	Minimum	Maximum	Stdev
tRNA	0.50	0.25	0.97	0.11
5s rRNA	0.63	0.40	0.96	0.12
RNase P RNA	0.65	0.41	0.99	0.10
SRP RNA	0.42	0.24	1	0.13

Prediction Stratified by Sequence Percent Identity

Table S8. Prediction accuracy for secondary structure prediction measure for sequence pairs stratified by pairwise sequence identity (identical nucleotides/aligned nucleotides). SRP RNA and RNase P RNA sequence alignments are acquired from databases (7,8). tRNA and 5S rRNA sequence alignments are predicted by inputting all the sequences into the ClustalW webserver (9). The identity range 0-20% does not include any sequence pairs, therefore it is not shown in the table.

Sensitivity/PPV	Dynalign II	Dynalign II w/o DI	Dynalign	Fold	Number of Sequence Pairs
20%≤Sequence Identity<40%					
tRNA	0.880/0.886	0.846/0.854	0.832/0.845	0.798/0.797	124
5S rRNA	0.939/0.939	0.939/0.939	0.939/0.925	0.364/0.308	1
RNase P RNA					0
SRP RNA	0.630/0.643	0.580/0.572	0.560/0.565	0.630/0.593	134
40%≤Sequence Identity<60%					
tRNA	0.916/0.921	0.869/0.879	0.853/0.861	0.799/0.769	531
5S rRNA	0.909/0.819	0.909/0.819	0.897/0.809	0.708/0.609	84
RNase P RNA	0.619/0.655	0.594/0.633	0.572/0.637	0.600/0.580	42
SRP RNA	0.759/0.778	0.767/0.767	0.757/0.761	0.694/0.670	57
60%≤Sequence Identity<80%					
tRNA	0.947/0.955	0.934/0.941	0.931/0.940	0.812/0.775	112
5S rRNA	0.920/0.846	0.920/0.846	0.920/0.841	0.751/0.661	82
RNase P RNA	0.659/0.688	0.660/0.681	0.617/0.668	0.612/0.577	117
SRP RNA	0.818/0.799	0.823/0.813	0.813/0.816	0.678/0.644	16
80%≤Sequence Identity<100%					
tRNA	0.956/0.992	0.956/0.992	0.908/0.936	0.894/0.881	13
5S rRNA	0.940/0.833	0.940/0.833	0.937/0.821	0.757/0.654	23
RNase P RNA	0.615/0.627	0.610/0.622	0.595/0.627	0.522/0.504	10
SRP RNA	0.604/0.610	0.623/0.635	0.596/0.609	0.546/0.531	7

P-values for Test of Statistical Significance of Improvements in Dynalign II over Dynalign and Fold

Table S9. One tail p-value for paired t-test for statistical significance of the improvement of Dynalign II over Dynalign (10).

	Sensitivity	PPV
5S rRNA	2×10^{-2}	4×10^{-4}
tRNA	2×10^{-12}	2×10^{-12}
RNase P RNA	7×10^{-9}	4×10^{-3}
SRP RNA	5×10^{-6}	4×10^{-7}

Table S10. One tail p-value for paired t-test for statistical significance of the improvement of Dynalign II over Fold (10).

	Sensitivity	PPV
5S rRNA	2×10^{-12}	2×10^{-12}
tRNA	2×10^{-12}	2×10^{-12}
RNase P RNA	1×10^{-6}	6×10^{-17}
SRP RNA	3×10^{-2}	1×10^{-8}

References

1. Fu, Y., Sharma, G. and Mathews, D.H. (2014), Submitted.
2. Reuter, J.S. and Mathews, D.H. (2010) RNAstructure: software for RNA secondary structure prediction and analysis. *BMC Bioinformatics*, **11**, 129.
3. Xia, T., SantaLucia, J., Jr., Burkard, M.E., Kierzek, R., Schroeder, S.J., Jiao, X., Cox, C. and Turner, D.H. (1998) Thermodynamic parameters for an expanded nearest-neighbor model for formation of RNA duplexes with Watson-Crick base pairs. *Biochemistry*, **37**, 14719-14735.
4. Mathews, D.H., Sabina, J., Zuker, M. and Turner, D.H. (1999) Expanded sequence dependence of thermodynamic parameters improves prediction of RNA secondary structure. *J Mol Biol*, **288**, 911-940.
5. Mathews, D.H. and Turner, D.H. (2002) Dynalign: an algorithm for finding the secondary structure common to two RNA sequences. *J Mol Biol*, **317**, 191-203.
6. Juhling, F., Morl, M., Hartmann, R.K., Sprinzl, M., Stadler, P.F. and Putz, J. (2009) tRNAdb 2009: compilation of tRNA sequences and tRNA genes. *Nucleic Acids Res*, **37**, D159-162.
7. Brown, J.W. (1999) The Ribonuclease P Database. *Nucleic Acids Res*, **27**, 314.
8. Rosenblad, M.A., Larsen, N., Samuelsson, T. and Zwieb, C. (2009) Kinship in the SRP RNA family. *RNA Biol*, **6**, 508-516.
9. Larkin, M.A., Blackshields, G., Brown, N.P., Chenna, R., McGettigan, P.A., McWilliam, H., Valentin, F., Wallace, I.M., Wilm, A. and Lopez, R. (2007) Clustal W and Clustal X version 2.0. *Bioinformatics*, **23**, 2947-2948.
10. Xu, Z., Almudevar, A. and Mathews, D.H. (2012) Statistical evaluation of improvement in RNA secondary structure prediction. *Nucleic Acids Res*, **40**, e26.
