# Supplementary material for: Dynalign II: common secondary structure prediction for RNA homologs with domain insertions
Source: Nucleic Acids Res. 2014 Nov 21;42(22):13939–48. doi: 10.1093/nar/gku1172 (PMC4267632; doi:10.1093/nar/gku1172)
Supplement: SUPPLEMENTARY DATA [file supp_gku1172_nar-02021-z-2014-File012.zip › manual/GUI/html/Appendix_of_File_Formats.html]

RNAstructure GUI Help -- Appendix A: File Formats


|  |  |  |
| --- | --- | --- |
|  | RNAstructure GUI Help Appendix A: File Formats | - Contents - Index |
| **Sequence File Format**  A sequence must be saved as a text file with a SEQ extension. The file must have the following format:   1. Comment lines must be at the beginning of the file and must start with a semicolon. There needs to be at least one comment line in the file. 2. Any number of comments are allowed, but each line before the title must start with a semicolon. 3. After the comments, a title must be given and must be contained on one line. 4. The line after the title must start the sequence, entered from 5' to 3'. The sequence can be on any number of lines and can contain U or T (T's are translated into U's for RNA). Spaces are allowed in the sequence. Bases should generally be in capital letters. **Lowercase letters can be used to indicate a base that should be single-stranded in the predicted structure.** "XXXX" can be used in the sequence to indicate that some bases have been left out of the prediction. 5. Finally, the sequence must end in 1.  SAMPLE  ``` ; (first line of file) Comments must start with a semicolon. ; ; There can be any number of comments. A title must immediately follow on the next line and be on one line. AAA GCGG UUTGTT UTCUTaaTCTXXXXUCAGG1 								 ```     ---  **FASTA (Sequence) File Format**  Sequences can also be provided to RNAstructure in FASTA format. For FASTA, the first line, a title line, needs to start with ">". Subsequent lines should only contain sequence and whitespace, which is ignored. **Lowercase nucleotides will be forced single stranded in structure prediction.**  Note that this file format can only be used in the JAVA GUI (Mac OS X and Linux).  SAMPLE  ``` > Title of Sequence AAA GCGG UUTGTT UTCUTaaTCTXXXXUCAGG UUA GCCG UUTGTT UTCUTaaTCTGGG 								 ```     ---  **CT File Format**  A CT (Connectivity Table) file contains secondary structure information for a sequence. These files are saved with a CT extension. When entering a structure to calculate the free energy, the following format must be followed.   1. Start of first line: number of bases in the sequence 2. End of first line: title of the structure 3. Each of the following lines provides information about a given base in the sequence. Each base has its own line, with these elements in order:    - Base number: index n    - Base (A, C, G, T, U, X)    - Index n-1    - Index n+1    - Number of the base to which n is paired. No pairing is indicated by 0 (zero).    - Natural numbering. RNAstructure ignores the actual value given in natural numbering, so it is easiest to repeat n here.   The CT file may hold multiple structures for a single sequence. This is done by repeating the format for each structure without any blank lines between structures.  The CT file format is such that any files generated by RNAstructure are compatible with mfold/Unafold (available from Michael Zuker), and many other software packages.  SAMPLE  ```   300  ENERGY = 7.0  example     1 G       0    2   22    1     2 G       1    3   21    2     3 G       2    4   20    3     4 C       3    5   19    4     5 G       4    6    0    5     6 A       5    7    0    6     7 A       6    8    0    7     8 U       7    9   18    8     9 U       8   10   17    9    10 G       9   11   16   10 (structure continues to next structure...)   300  ENERGY = 6.2  example     1 G       0    2    0    1     2 G       1    3    0    2     3 G       2    4   20    3     4 C       3    5   19    4     5 G       4    6    0    5     6 A       5    7    0    6     7 A       6    8    0    7     8 U       7    9   18    8     9 U       8   10   17    9    10 G       9   11   16   10 (structure continues to next structure or end of file...) 								 ```     ---  **Constraint File Format**  Folding constraints are saved in plain text with a CON extension. These can be hand edited. For multiple entries of a specific type of constraint, entries are each listed on a separate line. When there is no constraint of a type, there are no lines required. Note that all specifiers, followed by "-1" or "-1 -1", are expected by RNAstructure. For all specifiers that take two arguments, it is assumed that the first argument is the lower base pair number. The file format is as follows:   ``` DS: XA -1 SS: XB -1 Mod: XC -1 Pairs: XD1 XD2 -1 -1 FMN: XE -1 Forbids: XF1 XF2 -1 -1 							 ```  - XA: Nucleotides that will be double-stranded - XB: Nucleotides that will be single-stranded (unpaired) - XC: Nucleotides accessible to chemical modification - XD1, XD2: Forced base pairs - XE: Nucleotides accessible to FMN cleavage - XF1, XF2: Prohibited base pairs  SAMPLE  ``` DS: 15 25 76 -1 SS: 17 18 20 35 -1 Mod: 2 15 -1 Pairs: 16 26 -1 -1 FMN: -1 Forbids: 15 27 -1 -1 								 ```     ---  **SHAPE Reactivity File Format**  The file format for SHAPE reactivity comprises two columns. The first column is the nucleotide number, and the second is the reactivity.  Nucleotides for which there is no SHAPE data can either be left out of the file, or the reactivity can be entered as less than -500. Columns are separated by any white space.  Note that there is no header information. Nucleotides 1 through 10 have no reactivity information. Nucleotide 11 has a normalized SHAPE reactivity of 0.042816. Nucleotide 12 has a normalized SHAPE reactivity of 0, which is NOT the same as having no reactivity when using the pseudo-energy constraints.  By default, RNAstructure looks for SHAPE data files to have the file extension SHAPE, but any plain text file can be read.  SAMPLE  ``` 9    -999 10   -999 11   0.042816 12   0 13   0.15027 14   0.16201 								 ```     ---  **List File Format**  List files have a LIS extension. This file contains any number of sequences of any length or nucleic acid, each on its own line.  SAMPLE  ``` CUGAGCCAAG GGGCUCAACG GGCGUGAGAAAC 								 ```  Alignment File Format Alignment files are plain text. They are formatted as a nucleotide in the first sequence immediately followed by the nucleotide in the second sequence it's aligned to, separated by a space. Only one alignment pair can be on each line, and the last line of the file must be "-1 -1".  SAMPLE  ``` 10 12 11 13 -1 -1 								 ``` | | |
| Visit The Mathews Lab RNAstructure Page for updates and latest information. | | |
