# Supplementary material for: Dynalign II: common secondary structure prediction for RNA homologs with domain insertions
Source: Nucleic Acids Res. 2014 Nov 21;42(22):13939–48. doi: 10.1093/nar/gku1172 (PMC4267632; doi:10.1093/nar/gku1172)
Supplement: SUPPLEMENTARY DATA [file supp_gku1172_nar-02021-z-2014-File012.zip › manual/GUI/html/Appendix_of_Thermodynamic_File_Details.html]

RNAstructure GUI Help -- Appendix B: An explanation of the thermodynamic parameters used by RNAstructure


|  |  |  |
| --- | --- | --- |
|  | RNAstructure GUI Help Appendix B: An explanation of the thermodynamic parameters used by RNAstructure | - Contents - Index |
| There are nineteen files of free energies for RNA-RNA interactions. The derivation of these parameters is in Mathews, Disney, Childs, Schroeder, Zuker, and Turner (2004). The use of these parameters is explained by the Nearest Neighbor Database (NNDB). Here is a brief summary of what the files contain:  coaxial.dat  The free energy of coaxial stacking for two helixes with no intervening unpaired nucleotides. When applied, the top nucleotides are the continuous sequence and the bottom nucleotides are interrupted by the rest of the multibranch loop or exterior loop.  coaxstack.dat  The first of the tables for coaxial stacking with an intervening mismatch. This is the stack with the open backbone.  dangle.dat  Presents the free energy of dangling ends (5' or 3'). These energies are used in multibranch and exterior loops.  hexaloop.dat  A lookup table for hairpins of six nucleotides.  int11.dat  The lookup table for single mismatches.  int21.dat  A lookup table for the free energy of a 2x1 internal loop. The values presented are the total stability of the loop, including the entropic penalty and the terminal stack bonus.  int22.dat  A lookup table for the free energy of a 2x2 internal loop analogous to the 2x1 internal loop.  loop.dat  A table which contains the entropic penalty for closing hairpin, internal, and bulge loops. Each has its own penalty based on length.  miscloop.dat  Contains miscellaneous loop parameters.  stack.dat  Contains the nearest neighbor parameters for stacking in a helix.  tloop.dat  The tetraloop bonus table. For hairpin loops with four unpaired nucleotides, this table is consulted. If the sequence (starting with the 5' last paired nucleotide and finishing with its 3' paired nucleotide) appears in the table, the bonus is applied to the hairpin's stability.  triloop.dat  A lookup table for hairpin loops of three. It is applied analogously to the tetraloop table in Tloop.dat.  tstack.dat  For terminal mismatch stacking in exterior loops, i.e. loops that contain the ends of the sequence.  tstackcoax.dat  The second of the tables for coaxial stacking with an intervening mismatch. This is the stack with the continuous backbone.  tstackh.dat  Gives the free energy of stacking for a terminal mismatch in a hairpin loop.  tstacki.dat  Gives the free energy of stacking for a terminal mismatch in an internal loop.  tstacki23.dat  Used for terminal mismatch stacking in internal loops of size 2x3 unpaired nucleotides (and 3x2).  tstacki1n.dat  Used for terminal mismatch stacking in internal loops of size 1xn nucleotides where n>2.  tstackm.dat  Used for terminal stacking in a multibranch loop.  The above \*.dat files contain standard free energies at 37°C in kcal/mol.   ---  OligoWalk thermodynamic parameters are specified in two ways. First, special provisions must be taken for the DNA option (see below). Second, for the Tm calculation in OligoWalk, the DH and DS parameters are stored in three files:   - helix.dat - stack.ds - stack.dh  ---  For calculations with Dynalign, a separate miscloop.dat table, called *dynalignmiscloop.dat*, is consulted.   ---  Enthalpy change parameters are also now provided to make predictions at temperatures other than 37°C. These files are similar to those used for free energy change, but are terminated with the extension "DH."   ---  DNA-DNA parameters are used if the DNA option is selected in oligo walk or if DNA is folded. The DNA files are analogous to the RNA files, but the file names start with the letters "dna."   ---  If the DNA option is selected in OligoWalk or OligoScreen, DNA-RNA parameters are used. They are derived from Sugimoto, Nakano, Katoh, Matsumura, Nakamuta, Ohmichi, Yoneyama, & Sasaki. These are contained in *stackdr.dat*. | | |
| Visit The Mathews Lab RNAstructure Page for updates and latest information. | | |
