# Supplementary material for: Dynalign II: common secondary structure prediction for RNA homologs with domain insertions
Source: Nucleic Acids Res. 2014 Nov 21;42(22):13939–48. doi: 10.1093/nar/gku1172 (PMC4267632; doi:10.1093/nar/gku1172)
Supplement: SUPPLEMENTARY DATA [file supp_gku1172_nar-02021-z-2014-File012.zip › manual/GUI/html/Available_Modules_for_Prediction_and_Calculation.html]

RNAstructure GUI Help -- Available Modules for Prediction and Calculation


|  |  |  |
| --- | --- | --- |
|  | RNAstructure GUI Help Available Modules for Prediction and Calculation | - Contents - Index |
| **Break RNA Pseudoknots**  This item, unique to RNA, breaks pseudoknots in an RNA secondary structure, leaving a pseudoknot-free structure. See "Break RNA Pseudoknots."  **Dot Plot**  This item allows the display of a dot plot using one of many save file types generated from various manipulations of a sequence. See "Dot Plot."  **Draw**  This item will draw any structure saved in the CT file format. See "Draw."  **Dynalign**  This item opens the Dynalign tool, which will find the sequence alignment and the lowest free energy common structure common to two sequences. See "Dynalign."  **Efn2**  This item will determine the free energy of a secondary structure saved in the CT file format. See "Efn2."  **Fold Single Strand**  This item will open a folding window. This is the traditional structure prediction algorithm that finds lowest free energy structures. See "Fold."  **Fold Bimolecular**  This item will predict the intermolecular secondary structures of two oligonucleotides. See "Fold -> Bimolecular Folding."  **Generate All Suboptimal Structures**  This item allows the prediction of all possible secondary structures within a specified energy increment of the lowest free energy structure. See "Generate All Suboptimal Structures."  **MaxExpect: Predict MEA Structure**  This item predicts maximum expected accuracy structures, which are structures composed of the most probable pairs. See "Maximum Expected Accuracy."  **Multilign**  This item predicts common secondary structures for multiple sequences (more than two). See "Multilign."  **OligoScreen**  This item opens the OligoScreen tool, which determines thermodynamic parameters for a set of oligonucleotides. See "OligoScreen."  **OligoWalk**  This item opens the OligoWalk tool, which can help to determine an oligomer that will hybridize tightly to a target. See "OligoWalk."  **Partition Function**  This item uses a partition function calculation to predict base pair probabilities for any canonical pair in the sequence. See "Partition Function."  **Partition Function Bimolecular**  This item uses a partition function calculation to predict base pair probabilities for base pairs between two sequences. See "Partition Function -> Bimolecular Partitioning."  **ProbKnot: Predict Structures Including Pseudoknots**  This item will predict a maximum expected accuracy structure, including pseudoknots, from a partition function save file, and save the result as a CT file. See "ProbKnot."  **Refold**  This item can be used to predict a different set of suboptimal secondary structures from a previously folded sequence or calculation. See "Refold."  **Stochastic Sampling**  This item generates a sample of secondary structures for a sequence, drawn from the complete folding ensemble. See "Stochastic Sampling."  **TurboFold**  This item predicts common secondary structures for multiple sequences (more than two) using base pairing probabilities. See "TurboFold." | | |
| Visit The Mathews Lab RNAstructure Page for updates and latest information. | | |
