# Supplementary material for: Dynalign II: common secondary structure prediction for RNA homologs with domain insertions
Source: Nucleic Acids Res. 2014 Nov 21;42(22):13939–48. doi: 10.1093/nar/gku1172 (PMC4267632; doi:10.1093/nar/gku1172)
Supplement: SUPPLEMENTARY DATA [file supp_gku1172_nar-02021-z-2014-File012.zip › manual/GUI/html/Break_RNA_Pseudoknots.html]

RNAstructure GUI Help -- Break RNA Pseudoknots


|  |  |  |
| --- | --- | --- |
|  | RNAstructure GUI Help Break RNA Pseudoknots | - Contents - Index |
| This is a module unique to RNA, which breaks pseudoknots in an existing structure to determine the lowest free energy structure without pseudoknots.  **Breaking Knots in a Pseudoknotted Structure**   1. Click the button labeled "Input CT File." A dialog box will open to get the name of a CT file (which must contain pseudoknots). 2. A default name is provided for the output file free of pseudoknots. This can be changed by clicking the "Output CT File" button. 3. Select the way pseudoknots should be broken, if desired. This choice is available only in the JAVA GUI. In the Windows GUI, energy is always minimized. 4. Press the "Start" button. | | |
| Visit The Mathews Lab RNAstructure Page for updates and latest information. | | |
