# Supplementary material for: Dynalign II: common secondary structure prediction for RNA homologs with domain insertions
Source: Nucleic Acids Res. 2014 Nov 21;42(22):13939–48. doi: 10.1093/nar/gku1172 (PMC4267632; doi:10.1093/nar/gku1172)
Supplement: SUPPLEMENTARY DATA [file supp_gku1172_nar-02021-z-2014-File012.zip › manual/GUI/html/Contact.html]

RNAstructure GUI Help -- Contacting the Programmer


|  |  |  |
| --- | --- | --- |
|  | RNAstructure GUI Help Contacting the Programmer | - Contents - Index |
| To send notice of a bug, send e-mail to David Mathews (David\_Mathews@urmc.rochester.edu). In the subject of the message, please type "RNAstructure bug." In the body of the message, include a description of the bug and your full name. If a specific sequence or CT file was involved, it would be helpful if this was provided as an attachment. Bugs will be dealt with as soon as possible.  To ask for help, send e-mail to David Mathews (David\_Mathews@urmc.rochester.edu). In the subject of the message, please type "RNAstructure help." An attempt will be made to respond to all help questions.  To make a suggestion, send e-mail to David Mathews (David\_Mathews@urmc.rochester.edu). In the subject of the message, please type "RNAstructure suggestion." Suggestions are acted upon as time allows. | | |
| Visit The Mathews Lab RNAstructure Page for updates and latest information. | | |
