# Supplementary material for: Dynalign II: common secondary structure prediction for RNA homologs with domain insertions
Source: Nucleic Acids Res. 2014 Nov 21;42(22):13939–48. doi: 10.1093/nar/gku1172 (PMC4267632; doi:10.1093/nar/gku1172)
Supplement: SUPPLEMENTARY DATA [file supp_gku1172_nar-02021-z-2014-File012.zip › manual/GUI/html/Contents.html]

RNAstructure GUI Help -- Overview


|  |  |  |
| --- | --- | --- |
|  | RNAstructure GUI Help  Overview: Version 5.6 | - Contents - Index |
| General Help  - Introduction - Index - What's New - Secondary Structure Prediction Algorithm - Overview of Main Menus  Nucleic Acid Analysis Tools  - Sequence Editor - Available Modules for Prediction and Calculation - - Break RNA Pseudoknots   - Dot Plot   - Draw   - Dynalign   - Efn2 (Energy Function 2)   - Fold   - Generate All Suboptimal Structures   - MaxExpect: Predict Maximum Expected Accuracy Structure   - Multilign   - OligoScreen   - OligoWalk   - Partition Function   - ProbKnot: Predict Secondary Structure Including Pseudoknots   - Refolding   - Stochastic Sampling   - TurboFold - Tips and Techniques for Using Modules  Appendices  - Appendix A: File Formats - Appendix B: Thermodynamic Parameters Used by RNAstructure  Contacting the ProgrammerReferences | | |
| Visit The Mathews Lab RNAstructure Page for updates and latest information. | | |
