# Supplementary material for: Dynalign II: common secondary structure prediction for RNA homologs with domain insertions
Source: Nucleic Acids Res. 2014 Nov 21;42(22):13939–48. doi: 10.1093/nar/gku1172 (PMC4267632; doi:10.1093/nar/gku1172)
Supplement: SUPPLEMENTARY DATA [file supp_gku1172_nar-02021-z-2014-File012.zip › manual/GUI/html/Dot_Plot.html]

RNAstructure GUI Help -- Dot Plot


|  |  |  |
| --- | --- | --- |
|  | RNAstructure GUI Help Dot Plot | - Contents - Index |
| **Dot Plot Overview** RNAstructure can create several types of dot plots. The Dot Plot module produces an energy dot plot for a sequence. This shows, for each possible pair, the lowest free energy for a structure that contains that pair. Energy dot plots provide a picture of all alternative secondary structures within a given free energy increment of the lowest free energy structure. A large number of dots are displayed, when a small energy increment is chosen, suggests a less well-defined predicted secondary structure. Regions with many competing base pairs may be involved in alternative secondary structures or may be regions for which secondary structure prediction is inaccurate.  Note that two other types of dot plots can be created by RNAstructure, parition function dot plots and Dynalign dot plots (both below), but neither of these are made by the Dot Plot module.   ---  **Folding Dot Plot**  Folding dot plots illustrate base pairing energies read from a CT file. To display a folding dot plot, choose "File -> Dot Plot." Thse require save files from prior Fold calculations.   ---  **Partition Function Dot Plot**  Partition function dot plots display the probabilities of pairs, as predicted by the partition function. Dots are displayed as -log10(probability). The default view shows all possible pairs of any probability.  These dot plots are created when a partition function calculation is run.  To output base pair probabilities to a plain text file, choose "Output Plot -> Write to Dot Plot File." This will open a save dialog box in which the save file name can be specified.  To output the plot as a Postscript image file, choose "Output Plot -> Write to Postscript Image File."  Dot plots of previously calculated partition function calculations can be displayed by choosing "File -> Dot Plot Partition Function." This opens a dialog box for choosing the partition function save file. After choosing the save file, the dot plot is displayed.   ---  **Dynalign Dot Plot**  For Dynalign dot plots, a separate energy dot plot is generated for each of the two sequences involved. To display a Dynalign dot plot, choose "File -> Dot Plot Dynalign." This requires a save file from a prior Dynalign calculation.   ---  **Changing the Dot Plot Appearance**  The dot plot will be initially shown sized to the window in which it appears. To zoom to a specific region, the menu item "Draw -> Zoom" can be chosen to open a dialog box from which the zoom level can be adjusted. Alternatively, pressing the up or down arrow keys on the keyboard will zoom in or out, respectively. The scroll bars or arrow keys can be used to adjust the portion of the plot chosen.  Each color dot indicates an energy increment that is indicated by a key beneath the plot. The number of colors used in the plot can be adjusted from 3 to 15 by selecting the "Draw -> Choose Colors" menu option.  When first drawn, the energy dot plot shows all base pairs. To restrict the display to a specific energy increment, choose "Draw -> Plot Range". Also, note that the range of energies can be reset to show all pairs by clicking "Reset Range" in the dialog box. It is often helpful to limit the plot range to energies close to the lowest free energy (for example, within 10%) to focus on only the low energy competing structures.  To get the exact energy of any displayed base pair, click on the point in the plot with the left mouse button. The pair and its energy are displayed in a field above the plot. | | |
| Visit The Mathews Lab RNAstructure Page for updates and latest information. | | |
