# Supplementary material for: Dynalign II: common secondary structure prediction for RNA homologs with domain insertions
Source: Nucleic Acids Res. 2014 Nov 21;42(22):13939–48. doi: 10.1093/nar/gku1172 (PMC4267632; doi:10.1093/nar/gku1172)
Supplement: SUPPLEMENTARY DATA [file supp_gku1172_nar-02021-z-2014-File012.zip › manual/GUI/html/Draw.html]

RNAstructure GUI Help -- Draw


|  |  |  |
| --- | --- | --- |
|  | RNAstructure GUI Help Draw | - Contents - Index |
| **How to display an RNA secondary structure**   1. Select "File -> Draw." 2. Select an existing CT file to draw from the open dialog.   Note that, currently, structures without pseudoknots are drawn using the conventional collapsed format where paired nucleotides are close. Structures with pseudoknots are drawn using a circular format.  Note also that the JAVA GUI (but not the Windows GUI) contains an option to draw nucleotides circled or uncircled for clarity.   ---  **Scrolling**  If the structure is larger than the window, scroll bars appear at the edge of the window. The arrow keys can also be used to navigate around the screen.   ---  **Choosing a Structure**  If the CT file contains more than one structure, you may select which to view. On the menu, select "Draw -> Structure Number." If clicking does not change the structure, then the CT file contains only one structure.  Structures can also be chosen from the keyboard without using the menu option. Press the control-down arrow or control-up-arrow to change the current structure to a more suboptimal structure. Pressing the control-right-arrow key will change to a more optimally scoring structure. On Macintosh systems, structures can also be chosen by pressing the command key and the appropriate arrow.  Limitations of the secondary structure prediction algorithm may cause the order in which structures are displayed to be misleading. The output structures are sorted according to the lowest free energy, but structure #1, although predicted to be most optimal, might not be the actual structure.   ---  **Zooming**  Select "Structure -> Zoom."  Zooming can also be performed from the keyboard without using the menu options. Press the control-down arrow or control-up-arrow to zoom in or out, respectively. On Macintosh systems, zooming can also be done by pressing the command key and the appropriate arrow.   ---  **Printing**  Select "File -> Print." Choose desired printer and hit OK.   ---  **Copying**  On Windows, the image of the currently displayed structure can be copied to the clipboard by selecting "Edit->Copy" or by typing control-C.   ---  **Color Annotation of Additional Information**  The drawing can be annotated with additional information (by the form of color coding) in one of two ways. Selecting either of the menu options defined below will open a dialog box which will allow the appropriate type of file to be selected.   1. Base Pairing Probabilities:     For paired nucleotides, the probability is that of being in the specified pair. For single-stranded nucleotides, the probability is that of being single-stranded. The higher the probability, the greater the confidence in the prediction.     If the partition function has been used to predict base pairing probabilities for the sequence, show the color annotation by clicking "Annotations -> Add Probability Annotation."     In JAVA, the color key used for annotation is automatically displayed below the structure when necessary. In Windows, the color key can be displayed by clicking "Annotations -> Show Color Annotation Key." 2. SHAPE Data:     Nucleotides with higher SHAPE reactivity are more likely to be single-stranded.     If SHAPE constraints were added to the sequence prior to folding, display the data by clicking "Annotations -> Add SHAPE Annotation."     In JAVA, the color key used for annotation is automatically displayed below the structure when necessary. In Windows, the color key can be displayed by clicking "Annotations -> Show SHAPE Annotation Key."   Note that base pairing data and SHAPE data cannot be displayed together on a drawing; for the sake of clarity, they are always displayed separately.   ---  **Publication Quality Output**  There are several methods for producing publication quality figures from the RNAstructure results.  In general, the Draw module alone will not produce publication quality images of secondary structures. The program XRNA, created and distributed by the Santa Cruz RNA Center, is one of several tools for drawing RNA secondary structures and producing high quality output. This program is available for download from http://rna.ucsc.edu/rnacenter. It is written in Java and will run on most computer architectures. The draw module from RNAstructure will write .txt helix input files that are read by XRNA to describe the location of helixes. When displaying a secondary structure, this is accessed by "Draw -> Write to Helix (Text) File."  In JAVA only (Macintosh OS X and Linux), images can be exported to either Postscript or SVG files. Both structures and dot plots can be exported with their associated menu commands, under the "Draw" or "Output Plot" menus, respectively. Structures, in particular, can be exported with color annotation.  A final alternative is to print the image using a pdf writer instead of a printer to make a pdf. The pdf can then be edited using another program. | | |
| Visit The Mathews Lab RNAstructure Page for updates and latest information. | | |
