# Supplementary material for: Dynalign II: common secondary structure prediction for RNA homologs with domain insertions
Source: Nucleic Acids Res. 2014 Nov 21;42(22):13939–48. doi: 10.1093/nar/gku1172 (PMC4267632; doi:10.1093/nar/gku1172)
Supplement: SUPPLEMENTARY DATA [file supp_gku1172_nar-02021-z-2014-File012.zip › manual/GUI/html/Dynalign.html]

RNAstructure GUI Help -- Dynalign


|  |  |  |
| --- | --- | --- |
|  | RNAstructure GUI Help Dynalign | - Contents - Index |
| Dynalign is a tool that will find the lowest free energy sequence alignment and secondary structure common to two unaligned sequences. The input is two sequences. The output is two structures (saved in a CT file) and an alignment saved as plain text.  Dynalign uses the mutual information of the two sequences to constrain secondary structure prediction. This can result in a large improvement in the accuracy of secondary structure prediction. The algorithm generates an alignment of the two sequences, but does not depend on sequence identity. See "References" for more details.  Dynalign can predict a set of low energy structures and alignments, called suboptimal structures. A set of parameters (explained below) are used to define how many suboptimal structures to generate and how different from each other the suboptimal structures should be.   ---  **To predict the common structure and alignment with Dynalign**   1. Click the button labeled "Sequence File 1" and provide the name of the first SEQ file. 2. Click the button labeled "Sequence File 2" and provide the name of the second SEQ file. 3. Default names have been entered for CT File 1, CT File 2, and Alignment File. If desired, these names can be changed by clicking the corresponding buttons. 4. Choose whether single base pair inserts will be allowed into a single structure by checking or unchecking the box. 5. Default parameters have also been entered for suboptimal structure generation. These can be modified at this time. An explanation of these parameters can be found below. 6. A Dynalign save file (DSV file) can be generated by checking the Generate Save File checkbox. The save file is needed for making Dynalign dot plots. 7. Enter any constraints on the Dynalign calculation from the Force menu, and/or change the calculation temperature, if desired. (See "Tips and Techniques" for details.) 8. Nucleotides can be forced to align in the alignment using the Constraints for Alignment menu. Constraints can be written to a file for reuse or read from a previously written file (See "Tips and Techniques"). 9. By default, Dynalign currently predicts a sequence alignment of the two sequences using an HMM model. Posterior probabilities are used to limit the search space used by Dynalign. 10. Click "Start" to begin the Dynalign algorithm. This calculation may take some time. A progress bar opens to indicate the approximate progress of the calculation. 11. When complete, windows will open to display the structures predicted for each sequence. The alignment file is plain text and can be viewed with any text editor, such as Wordpad, TextEdit, or Emacs. A dot plot can also be displayed if a save file was generated.  ---  **Parameters controlling the prediction of suboptimal structures**  Max % Energy Difference  The maximum percent change in energy between the lowest free energy structure and the most suboptimal structure. Increasing this percentage can increase the number of structures generated.  Max Number of Structures  An absolute limit on the number of generated suboptimal structures.  Window Sizes Note that larger windows require that the suboptimal structures be relatively more different from each other.  Structure Window Size  In each suboptimal structure, there must be at least one new base pair that is separated from all base pairs in other suboptimal structures by *window* nucleotides. Once a base pair is formed between nucleotides *i* and *j* in a suboptimal structure, the square region of base pairs from *i*-*window* to *i*+*window* paired to *j*-*window* to *j*+*window* is marked as represented. Subsequent suboptimal structures must have at least one pair outside of the marked region.  Alignment Window Size  The alignment window requires that each suboptimal alignment be different from other alignments by having two aligned nucleotides at least *window* nucleotides from other alignments. The smaller the windows (to the minimum value of zero) the more suboptimal structures that can be generated and the more similar the suboptimal structures are to each other. | | |
| Visit The Mathews Lab RNAstructure Page for updates and latest information. | | |
