# Supplementary material for: Dynalign II: common secondary structure prediction for RNA homologs with domain insertions
Source: Nucleic Acids Res. 2014 Nov 21;42(22):13939–48. doi: 10.1093/nar/gku1172 (PMC4267632; doi:10.1093/nar/gku1172)
Supplement: SUPPLEMENTARY DATA [file supp_gku1172_nar-02021-z-2014-File012.zip › manual/GUI/html/Efn2.html]

RNAstructure GUI Help -- Efn2 (Energy Function 2)


|  |  |  |
| --- | --- | --- |
|  | RNAstructure GUI Help Efn2 (Energy Function 2) | - Contents - Index |
| This module provides energy calculations for a structure, given its CT file. When calculating the energy of structures in a CT file, the OUT file lists the structure number followed by the calculated energy. **Note that efn2 does not support structures with pseudoknots.** The module will show an error if a CT file structure containing pseudoknots is given as input.  **How to Calculate the Free Energy of a Structure**   1. Click the button labeled "CT File." A dialog box will open to get the name of a CT file. 2. A default name is provided for the OUT file. This can be changed by clicking the "OUT File" button. 3. If desired, the temperature can be changed. (See "Tips and Techniques.") 4. Press the "Start" button. 5. The OUT file is written as a plain text file that can be opened with any text editor. 6. A more detailed OUT file, describing the energies within structures, can be created by checking the "Create Thermodynamic Details File" box. | | |
| Visit The Mathews Lab RNAstructure Page for updates and latest information. | | |
