# Supplementary material for: Dynalign II: common secondary structure prediction for RNA homologs with domain insertions
Source: Nucleic Acids Res. 2014 Nov 21;42(22):13939–48. doi: 10.1093/nar/gku1172 (PMC4267632; doi:10.1093/nar/gku1172)
Supplement: SUPPLEMENTARY DATA [file supp_gku1172_nar-02021-z-2014-File012.zip › manual/GUI/html/Fold.html]

RNAstructure GUI Help -- Fold


|  |  |  |
| --- | --- | --- |
|  | RNAstructure GUI Help Fold | - Contents - Index |
| The Fold module provides the basic implementation of RNA secondary structure prediction. Note that this implementation cannot predict pseudoknots.   ---  **How to predict secondary structure of a single strand**   1. Start the module by choosing "Fold Single Strand." This option is supported for both RNA and DNA. Click on the button labeled "Sequence File." A dialog box will open to get the name of a file containing a sequence. This sequence must be a SEQ file. Other sequences can be converted by the sequence editor. 2. After accepting the name of a file, default values are filled in for the remaining fields. These can be changed by clicking the button labeled "CT File", or by changing the values in the "Maximum % Energy Difference," "Maximum # of Structures," and "Window Size" boxes. 3. A checkbox next to "Generate Save File" should be checked in order to save the secondary structure prediction information. This file can be used later to generate a different set of suboptimal secondary structures or to display an energy dot plot for the secondary structure. The save file is named the same as the CT file, but with a SAV extension. 4. Enter any constraints on the folding calculation from the Force menu, change the calculation temperature, edit the maximum internal/bulge loop size, or change the maximum pairing distance, if desired. (See "Tips and Techniques" for details.) 5. Click on the "Start" button. A dialog will open to indicate the progress of the fill algorithm, the most time consuming step in the process. 6. After the calculation is complete, a dialog box will open to present the option of drawing the calculated structures. Click "OK" to draw the structure, or "Cancel" to quit the module.   The folding procedure for a single strand can also be accessed by a button on the Quick Access Toolbar. This button supports folding of an RNA single strand only.   ---  **Options**  Three options are allowed in the "Fold" window. These concern the differences between, and number of, structures given in the output CT file after Efn2 rearrangement.  The algorithm outputs structures until either of the following first two criteria are reached. If only one structure meets the "Maximum Percent Energy Difference" criterion, then only one structure is generated, regardless of the "Maximum Number of Structures" setting.  Maximum Percent Energy Difference  Sets the percent difference from the lowest free energy allowed for the structures output. For example, if the lowest-free energy (ΔG°37) structure is -100 kcal/mol, and the Maximum Percent Energy Difference is 10, any structures with an energy of -90 kcal/mol or higher are rejected (higher means less negative).  Maximum Number of Structures  Sets an absolute upper limit on the number of structures that can be generated.  Window Size  This parameter controls how different the suboptimal structures must be from each other. A small window size allows very similar structures to be generated, while a larger window size requires them to be relatively more different.   ---  **Bimolecular Folding**  For both DNA and RNA, "Fold Bimolecular" allows for folding of two distinct strands. The folding of two strands functions in much the same way as folding of a single strand.  Double-stranded folding doesn't allow for the same wide range of constraints as single-stranded folding. The only constraint allowed for double-stranded folding is the prohibition or permission of intramolecular pairs (pairs within a distinct strand, rather than between the two separate strands). | | |
| Visit The Mathews Lab RNAstructure Page for updates and latest information. | | |
