# Supplementary material for: Dynalign II: common secondary structure prediction for RNA homologs with domain insertions
Source: Nucleic Acids Res. 2014 Nov 21;42(22):13939–48. doi: 10.1093/nar/gku1172 (PMC4267632; doi:10.1093/nar/gku1172)
Supplement: SUPPLEMENTARY DATA [file supp_gku1172_nar-02021-z-2014-File012.zip › manual/GUI/html/Generate_All_Suboptimal_Structures.html]

RNAstructure GUI Help -- Generate All Suboptimal Structures


|  |  |  |
| --- | --- | --- |
|  | RNAstructure GUI Help Generate All Suboptimal Structures | - Contents - Index |
| Two suboptimal secondary structure routines are available in RNAstructure. The first is based on the popular mfold program and provides a set of representative low free energy structures in addition to the lowest free energy structure. The second is based on the algorithm of Wuchty et al. and can generate all possible secondary structures for a (small) free energy increment above the lowest free energy structure.  **Predicting All Suboptimal Structures**   1. Choose "Generate All Suboptimal Structures" to open the module. 2. Choose the sequence by clicking the button labeled "Sequence File." 3. A default output file (CT file) is provided. That can be changed by clicking the button labeled "CT File." 4. Choose the upper energy limit for the predicted secondary structures. The program stops generating structures when it either reaches the maximum limit (in kcal/mol) or the maximum percent difference limit. This limit needs to be carefully chosen. There is an exponential increase in the number of predicted structures as the energy limit is increased in size. It is best to choose a conservative cutoff (perhaps <1 kcal/mol) for first calculations and then carefully increase this value. 5. Enter any constraints on the calculation from the Force menu, and/or change the calculation temperature, if desired. (See "Tips and Techniques" for details.) 6. Click "Start." When the program is complete, the option to display structures is given. Click "OK" to display the structures, or "Cancel" to close the module. | | |
| Visit The Mathews Lab RNAstructure Page for updates and latest information. | | |
