# Supplementary material for: Dynalign II: common secondary structure prediction for RNA homologs with domain insertions
Source: Nucleic Acids Res. 2014 Nov 21;42(22):13939–48. doi: 10.1093/nar/gku1172 (PMC4267632; doi:10.1093/nar/gku1172)
Supplement: SUPPLEMENTARY DATA [file supp_gku1172_nar-02021-z-2014-File012.zip › manual/GUI/html/Index.html]

RNAstructure GUI Help -- Index


|  |  |  |
| --- | --- | --- |
|  | RNAstructure GUI Help Index | - Contents - Index |
| A B C D E F G H I J K L M N O P Q R S T U V W X Y Z   ---  |  |  | | --- | --- | | A About RNAstructure  Accuracy  Acknowledgements  Algorithm  Alignment File Format  Annotating a Structure  Asking for Help  Audio Playback | N | | B Breaking Pseudoknots  Bulge Loop Size | O OligoScreen  OligoScreen Thermodynamic Parameters  OligoWalk  OligoWalk Calculations  OligoWalk Modes  OligoWalk Results  OligoWalk Thermodynamic Parameters  Outputting Base Pair Probabilities  Overview | | C Calculating Stochastic Samples  Calculation Modules  Changing Temperature of Calculations  Constraint File Format  Creating a Sequence  CT File Format | P Partition Function  Partition Function Bimolecular  Partition Function Dot Plots  Plot Range  Postscript Output  Precision  Predicting Base Pair Probabilities  Predicting Pseudoknots  Prediction Modules  Printing a Structure  Probability Annotation  ProbKnot  Pseudo-Energy Constraints (SHAPE)  Pseudoknots  Publication Quality Output | | D DH Thermodynamic Parameters  DNA Menu  DNA Thermodynamic Parameters  DNA-RNA Thermodynamic Parameters  Dot Plot Appearance  Dot Plot Color Selection  Dot Plot Module  Double Stranded Bases  Draw Module  Drawing a Structure  DS Thermodynamic Parameters  Dynalign Dot Plots  Dynalign Thermodynamic Parameters | Q Quick Access Toolbar | | E Editing a Sequence  Efn2 (Energy Function 2)  Enthalpy Thermodynamic Parameters | R Read While Typing  Refold  Refolding Dynalign Calculations  Refolding Structures  Rendering a Structure by Direction  Report a Bug  Resetting Constraints  Restoring Constraints  Reviewing Constraints  RNA Menu | | F FASTA File Format  File Formats  File Menu  Folding Dot Plots  Forbidding a Base Pair  Forcing a Base Pair  Forcing Double Stranded Bases  Forcing Single Stranded Bases  Formatting a Sequence  Free Energy Calculation  Free Energy Thermodynamic Parameters | S Saving Constraints  Sequence Entry Particulars  Sequence File Format  SHAPE Annotation  SHAPE Constraints  SHAPE File Format  Single Stranded Bases  Specifying Chemical Modification  Specifying FMN Cleavage  Stochastic Sampling  Support  Switching Between Structures  System Requirements | | G Generating Suboptimal Structures | T Thermodynamic Parameters  Toolbar | | H Hard Constraints (SHAPE)  Help Menu | U Using Calculation Constraints  Using OligoScreen  Using OligoWalk | | I Image Output  Internal Loop Size | V Variable Menus  Viewing an Entire Structure | | J | W | | K | X | | L List File Format  Loading a Sequence | Y | | M Making Suggestions  Maximum Expected Accuracy  Module List | Z Zooming a Structure | | | |
| Visit The Mathews Lab RNAstructure Page for updates and latest information. | | |
