# Supplementary material for: Dynalign II: common secondary structure prediction for RNA homologs with domain insertions
Source: Nucleic Acids Res. 2014 Nov 21;42(22):13939–48. doi: 10.1093/nar/gku1172 (PMC4267632; doi:10.1093/nar/gku1172)
Supplement: SUPPLEMENTARY DATA [file supp_gku1172_nar-02021-z-2014-File012.zip › manual/GUI/html/Introduction.html]

RNAstructure GUI Help -- Introduction


|  |  |  |
| --- | --- | --- |
|  | RNAstructure GUI Help Introduction | - Contents - Index |
| General Information The core of RNAstructure is a dynamic programming algorithm to predict RNA or DNA secondary structures from sequence based on the principle of minimizing free energy. The thermodynamic parameters used for RNA predictions are the latest available from the Turner laboratory. Several modules are provided to extend the capabilities of secondary structure prediction, including the prediction of a secondary structure common to two sequences, and to make this a user-friendly RNA folding program.  RNAstructure was originally written by David H. Mathews for Isis Pharmaceuticals and is made available for the RNA community by Isis. RNAstructure is now supported by U.S. National Institutes of Health grant #R01GM076485. It is available on the Mathews Lab Homepage.  Two other modern implementations of the dynamic programming algorithm for RNA secondary structure prediction are mfold/UNAfold, available on the World Wide Web at Michael Zuker's homepage, and the Vienna RNA package, maintained by Ivo Hofacker. The implementation of the thermodynamic parameters differs in multibranch and exterior loops, therefore some differences in the predicted lowest free energy structures are expected by different packages.  Research benefitting from this program should cite the journal references on which this program is based (See "References").   ---  Requirements RNAstructure can be run on Windows, Macintosh, or Linux systems.  On the Macintosh and on Linux, the program requires Java version 1.6 or higher. For best results, the implementation of Java obtained directly from Sun Microsystems should be used. Other implementations may cause aberrant behavior. The Sun implementation of Java can be found at the Java download pages.   ---  Contributors to Code David Mathews is the primary author of this program.  Stanislav Bellaousov contributed code for ProbKnot.  Jason Gloor contributed code for MEA structure prediction.  Arif Haramanci contributed the HMM alignment code used to reduce Dynalign's search space and the TurboFold algorithm.  Zhi Lu revised the free energy minimization and the partition function code to predict internal loops of any size in O(N3) time. He also contributed code for MEA structure prediction.  Jessica Reuter contributed the Java interface and shared libraries that allowed the program to be portable across multiple operating systems.  Paul Tymann, Glenn Katzen, Chris Connett, and Andrew Yohn, Computer Science, Rochester Institute of Technology, contributed changes that accelerated Dynalign.  Zhenjiang Xu wrote the code for Multilign.   ---  Special Thanks Thanks are deserved to those who helped with the creation and improvement of the program, including Marc Brower, Mark Burkard, Karina Burkard, Katie Deigan, Josh Diamond, Karim Fakhreddine, Sue Freier, Christine Hajdin, Arif Harmanci, Wayne Huggins, James Kim, Zhi John Lu, Olga Matveeva, Jeff McDowell, Jessica Reuter, Jeff Sabina, Gaurav Sharma, Steve Testa, Brent Townshend, Doug Turner, Rahul Tyagi, Kevin Weeks, Jacqueline Wyatt, and Michael Zuker. | | |
| Visit The Mathews Lab RNAstructure Page for updates and latest information. | | |
