# Supplementary material for: Dynalign II: common secondary structure prediction for RNA homologs with domain insertions
Source: Nucleic Acids Res. 2014 Nov 21;42(22):13939–48. doi: 10.1093/nar/gku1172 (PMC4267632; doi:10.1093/nar/gku1172)
Supplement: SUPPLEMENTARY DATA [file supp_gku1172_nar-02021-z-2014-File012.zip › manual/GUI/html/Maximum_Expected_Accuracy.html]

RNAstructure GUI Help -- Maximum Expected Accuracy


|  |  |  |
| --- | --- | --- |
|  | RNAstructure GUI Help Maximum Expected Accuracy | - Contents - Index |
| This module predicts structures with maximum and near maximum expected accuracy. These are structures that maximize pair probabilities.  The scoring function used is: score = gamma\*(sum of pair probabilities for pairs) + (sum of unpairing probabilities for unpaired nucleotides)  **Predicting Maximum Expected Accuracy Structures**   1. First choose a save file from a previous partition function calculation. Click the button labeled "Partition Function Save File." A dialog box will open to get the name of a PFS file. 2. A default name is provided for the output file that will contain the predicted structures. This can be changed by clicking the "CT File" button. 3. Change the weight given to pairs by editing the default value for "Gamma." The default is generally the best option. 4. Change suboptimal structure parameters by changing the appropriate values under "Suboptimal Structure Parameters." The maximum number of suboptimal structures is controlled by both the "Max % Score Difference" and the "Max Number of Structures." Structures are generated until either the max number of structures is reached or the scores differ by greater than Max % Score Difference from the optimal score. "Window" controls how different the structures are from each other. A higher Window will generate structures that are more different and a small Window (such as zero) will result in structures that are more subtly different. 5. Press the "Start" button. 6. When the calculation is complete, you will be asked whether to draw the predicted structure on the screen. | | |
| Visit The Mathews Lab RNAstructure Page for updates and latest information. | | |
