# Supplementary material for: Dynalign II: common secondary structure prediction for RNA homologs with domain insertions
Source: Nucleic Acids Res. 2014 Nov 21;42(22):13939–48. doi: 10.1093/nar/gku1172 (PMC4267632; doi:10.1093/nar/gku1172)
Supplement: SUPPLEMENTARY DATA [file supp_gku1172_nar-02021-z-2014-File012.zip › manual/GUI/html/Multilign.html]

RNAstructure GUI Help -- Multilign


|  |  |  |
| --- | --- | --- |
|  | RNAstructure GUI Help Multilign | - Contents - Index |
| Multilign is a tool that will find the lowest free energy secondary structure common to multiple (more than two) unaligned sequences and the sequence alignment that reflects the conserved structure. The input is more than two sequences. The output is an equivalent number of structures (saved as CT files) and an alignment saved as plain text.  Multilign uses the mutual information of the sequences to constrain secondary structure prediction. This information is applied in a progressive, iterative fashion, using Dynalign, to continually improve prediction. This can result in a large improvement in the accuracy of secondary structure prediction. The algorithm generates an alignment of the sequences, but does not depend on sequence identity. See "References" for more details.  Multilign can predict a set of low energy structures and alignments, called suboptimal structures. A set of parameters (explained below) are used to define how many suboptimal structures to generate and how different from each other the suboptimal structures should be.   ---  **To predict the common structures and alignment with Multilign**   1. Click the button labeled "Sequence File" and provide the name of a sequence file. 2. A default name has been entered for the CT File. If desired, this name can be changed by clicking the corresponding button. 3. Click the "ADD" button to add the sequence and CT files to the sequence set under consideration. Note that at least three sequences must be added for Multilign to run correctly. 4. Continue adding sequence and CT files to the sequence set in this manner until all desired input and output files have been specified. 5. If a sequence/CT pair was entered in error, it can be removed by clicking the "Delete Sequence" button. The number of the sequence to be removed can be found in the text box next to the button, and it is by default the last sequence. However, specific sequence/CT pairs can be deleted by changing this value. Only one sequence can be deleted at a time. 6. After the first sequence/CT pair has been added to the sequence set, a default file name for the multiple alignment file is given. To change this name, click the "Alignment File" button. 7. Choose whether single base pair inserts will be allowed into a single structure by checking or unchecking the box. 8. Choose whether pairwise Dynalign save files and alignments will be generated by clicking the box. These files will be generated in the same directory as the CT file associated with the index sequence (see below). So, for example, if the index sequence is the third one (3), the intermediate files will be generated in the same directory as the CT file for sequence 3. (Note that on Windows, the files must be generated.) 9. Default parameters have also been entered for suboptimal structure generation. These can be modified at this time. An explanation of these parameters can be found below. 10. The Multilign-specific Max Pairs parameter is variable, and changes depending on the number and content of sequences given. If a specific value should be used, that value should be input into the box **after** all sequence/CT pairs have been added. 11. Click "Start" to begin the Multilign algorithm. This calculation may take some time. A progress bar opens to indicate the approximate progress of the calculation. 12. When complete, windows will open to display the structures predicted for each sequence. The alignment file is plain text and can be viewed with any text editor, such as Wordpad, TextEdit, or Emacs.  ---   **Parameters controlling the prediction of suboptimal structures**  Max % Energy Difference  The maximum percent change in energy between the lowest free energy structure and the most suboptimal structure. Increasing this percentage can increase the number of structures generated.  Max Number of Structures  An absolute limit on the number of generated suboptimal structures.  Window Sizes Note that larger windows require that the suboptimal structures be relatively more different from each other.  Structure Window Size  In each suboptimal structure, there must be at least one new base pair that is separated from all base pairs in other suboptimal structures by *window* nucleotides. Once a base pair is formed between nucleotides *i* and *j* in a suboptimal structure, the square region of base pairs from *i*-*window* to *i*+*window* paired to *j*-*window* to *j*+*window* is marked as represented. Subsequent suboptimal structures must have at least one pair outside of the marked region.  Alignment Window Size  The alignment window requires that each suboptimal alignment be different from other alignments by having two aligned nucleotides at least *window* nucleotides from other alignments. The smaller the windows (to the minimum value of zero) the more suboptimal structures that can be generated and the more similar the suboptimal structures are to each other.  Iterations  The number of iterations for the calculation to go through.  Max Pairs  The maximum number of pairs allowed in structures.  Max DSV Change  The maximum percent change allowed during progressive comparisons. | | |
| Visit The Mathews Lab RNAstructure Page for updates and latest information. | | |
