# Supplementary material for: Dynalign II: common secondary structure prediction for RNA homologs with domain insertions
Source: Nucleic Acids Res. 2014 Nov 21;42(22):13939–48. doi: 10.1093/nar/gku1172 (PMC4267632; doi:10.1093/nar/gku1172)
Supplement: SUPPLEMENTARY DATA [file supp_gku1172_nar-02021-z-2014-File012.zip › manual/GUI/html/OligoScreen.html]

RNAstructure GUI Help -- OligoScreen


|  |  |  |
| --- | --- | --- |
|  | RNAstructure GUI Help OligoScreen | - Contents - Index |
| **Overview**  OligoScreen calculates the unimolecular and bimolecular folding free energies for a set of RNA or DNA oligonucleotides. It also calculates a hybridization free energy for those strands annealed to a complementary RNA target.  The OligoScreen parameters are a subset of those calculated by OligoWalk. They have been shown to correlate with antisense activity.  OligoScreen requires an input file in the LIS (List) format. See the "Appendix of File Formats" for detals on the LIS format.   ---  **Using OligoScreen**   1. To start OligoScreen, choose "File -> OligoScreen." 2. Click on "Oligomer List" to choose the LIS file with the input sequences. 3. An ouput filename will be automatically chosen, but can be changed by clicking the "Output Filename" button. 4. Specify whether the Oligonucleotides are DNA or RNA by clicking the appropriate button next to "DNA" or "RNA", respectively, in the "Oligomer Chemistry" box. 5. For an RNA sequence, both T and U in the input are interpreted as U. Similarly, choosing DNA causes T and U to be interpreted as T. 6. If desired, the temperature for prediction can be changed. (See "Tips and Techniques.") 7. Finally, start the calculation by clicking the "Start" button. 8. The output is written to the output file, which is plain text, tab delimited. | | |
| Visit The Mathews Lab RNAstructure Page for updates and latest information. | | |
