# Supplementary material for: Dynalign II: common secondary structure prediction for RNA homologs with domain insertions
Source: Nucleic Acids Res. 2014 Nov 21;42(22):13939–48. doi: 10.1093/nar/gku1172 (PMC4267632; doi:10.1093/nar/gku1172)
Supplement: SUPPLEMENTARY DATA [file supp_gku1172_nar-02021-z-2014-File012.zip › manual/GUI/html/OligoWalk.html]

RNAstructure GUI Help -- OligoWalk


|  |  |  |
| --- | --- | --- |
|  | RNAstructure GUI Help OligoWalk | - Contents - Index |
| This module provides a rapid search interface for picking out an oligonucleotide that binds strongly to its target, according to thermodynamic data. It not only calculates energy required to break target structure, but also structure due to oligonucleotide folding, if any.   ---   **Using OligoWalk**   1. Select "OligoWalk." 2. Choose the CT file for the desired target by clicking the "CT File" button. 3. A report File with a REP extension is automatically chosen. This can be changed by clicking the "Report File" button. 4. Choose the mode from the three possibilities. Most publications have used "Break Local Structure." 5. Choose the desired length of a binding oligonucleotide, and choose the oligonucleotide concentration. 6. Change the temperature of calculation, if desired. (See "Tips and Techniques" for details.) 7. Click the "Start" button to start the calculation. The progress of the calculation will be shown with a progress bar. 8. When calculation is finished, the OligoWalk results screen will open automatically.  ---   **Modes**  Three modes are available; these affect the speed and calculation type of free energy.   1. The default (and suggested mode for most use) is "Break Local Structure." In this mode, the target sequence is considered to lose base pairs wherever the oligo invades. The oligos may lose pairs in self structure, and gain pairs in oligo-target binding. All the oligos are folded to identify possible self structure. 2. For a lengthier calculation, select "Refold For Each Oligo." In this mode, the target is refolded for each possible oligo (hence it could take a while) in case the target may fold into new structure due to oligo binding. 3. The fastest mode (but only slightly faster than "Break Local Structure" is "Don't Use Structural Information." In this mode, the breaking of target structure is not considered. This may be useful if you have little confidence in the lowest free energy target structure. This mode can also be used with a sequence that has not been folded by selecting a sequence instead of a CT file. In most cases, it is preferable to include the free energy cost of the target structure.   While not explicitly a mode, the option "Include Target Suboptimal Structures" functions as a sort of fourth mode. This option uses all suboptimal structures in the target CT file to determine the free energy lost in the target. Each structure's free energy loss is weighted according to the free energy of the structure.   ---  **Results Screen**  The OligoWalk results screen contains three main parts. At the top, the current oligo number appears with the thermodynamics (ΔG) for this oligo, in kcal/mol. The oligo number also indicates the 5'-most base in the target that the oligo is designed to bind. The given ΔG's are as follows:   1. Overall ΔG: This is the net ΔG in kcal/mol of oligo-target binding, when all contributions are considered, including breaking target structure and oligo self-structure, if any. A more negative value indicates tighter binding. 2. Duplex ΔG (Binding ΔG): This is the ΔG of the oligo-target binding from unstructured states. 3. Break target ΔG: This number provides the energy penalty (hence it's usually positive) due to breaking of intramolecular target base pairs when oligo is bound. This free energy is always zero in "Don't Use Structural Information" mode. 4. Oligo-self ΔG: This provides the ΔG of intramolecular oligo structure. If it is zero, there is no stable intramolecular structure. A negative number indicates this self structure is stable, making for unfavorable oligo-target binding. 5. Oligo-oligo ΔG: This is the ΔG of intermolecular oligo structure. It is non-zero if one oligomer molecule can bind to another. A negative number indicates a stable oligo-oligo duplex, making for unfavorable oligo-target binding.   Tm: This is a melt temperature in degrees C for the duplex formation, i.e. the temperature at which half the target strands are bound with oligomer. It neglects structure in the oligo and target and assumes that oligomer concentration is in excess. An overview of this calculation is given below.  Below the thermodynamic data are five buttons. These are used to navigate through the rna sequence. Click "<" or ">" to move to the previous or next oligo. Click "<<" or ">>" to move back or forward by ten. Click "Goto..." to enter an oligo number to move to directly. Note that in the "Goto..." dialog box, you can click on "Most Stable" to select the highest affinity oligo and go there directly.  The middle part of the screen shows the current oligo (3'->5') bound to the target (5'->3'). Target bases appear red if they are paired in the folded target structure and are black otherwise. Target base numbers are given.  At the bottom, some ΔG values are displayed graphically. The default is to show binding ΔG in green and overall ΔG in blue (red for the currently shown oligo). Due to breaking of target and oligo structure, the blue bars are generally smaller than the green bars. Downward bars indicate negative ΔG and upward bars indicate positive ΔG. All bars start at zero energy.   - The currently graphed free energies can be changed by selecting the "Graph -> Free Energy" menu option. - Click on any bar in the graph to see the corresponding oligo. This bar will move to the center and be highlighted (red). - Click on any base in the target sequence to get information on the base, including its pair, if any, in the target structure before addition of the oligonucleotide. - Double click on the oligonucleotide to view oligonucleotide self-structure, if any.  ---  **Equilibria and Calculations**  *OligoWalk Equilibrium*  When designing an antisense oligonucleotide (oligomers) that will bind with high affinity, it is desirable to consider the structure of the target RNA strand and the antisense oligomer. Specifically, for an oligomer to bind tightly, it should be complementary to a stretch of target RNA that has little self-structure. Also, the oligomer should have little self-structure, either intramolecular or bimolecular. Breaking up any self-structure amounts to a binding penalty.  OligoWalk considers the following equilibrium:  In this reaction, O is the oligomer, T is the target, O-T is the oligomer-target complex. OF is self-structured oligomer either unimolecular (U) or bimolecular (B) and TF is self-structured target (unimolecular). Bimolecular target-target interactions are neglected because the concentration of target is low. OU is unfolded oligomer and TU is unfolded target in the region of oligomer complementarity. These structures are in equilibrium with each other, with equilibrium constants K1U, K1B, K2, and K3.  **The Tm Calculation in OligoWalk**  OligoWalk calculates a melt temperature for the duplex formation of antisense-target binding. This calculation neglects target structure and antisense oligonucleotide structure. Consider the equilibrium of:  where the random coil oligomer binds to random coil target with an equilibrium constant K. If we assume that:  **[OR.C.] >> [TR.C.]**  then the Tm will be the temperature at which half the target is bound or:  **[TR.C.] = [O-T] = [Target]Total/2**  Knowing that:  **ΔG = ΔH - TΔS and ΔG = -RT ln(K)**  Then:  where R is the gas constant and Tm is in K. OligoWalk converts Tm to degrees C. | | |
| Visit The Mathews Lab RNAstructure Page for updates and latest information. | | |
