# Supplementary material for: Dynalign II: common secondary structure prediction for RNA homologs with domain insertions
Source: Nucleic Acids Res. 2014 Nov 21;42(22):13939–48. doi: 10.1093/nar/gku1172 (PMC4267632; doi:10.1093/nar/gku1172)
Supplement: SUPPLEMENTARY DATA [file supp_gku1172_nar-02021-z-2014-File012.zip › manual/GUI/html/Overview_of_Main_Menus.html]

RNAstructure GUI Help -- Appendix A: Overview of Main Menus


|  |  |  |
| --- | --- | --- |
|  | RNAstructure GUI Help Overview of Main Menus | - Contents - Index |
| **File Menu**  The File menu holds all commands and modules that are not used specifically for one nucleic acid type or another. These commands and modules include opening a new sequence, refolding from save files, and accessing the OligoScreen module.  Unlike the rest of the menus present at startup, the contents of the File menu can change based on the topmost window displayed. Depending on the window displayed, the menu may also include save or print options.   ---   **RNA Menu**  The RNA menu holds all modules that are specifically targeted to use with RNA. Some of its modules have counterparts in the DNA menu, while others do not.   ---   **DNA Menu**  The DNA menu holds all modules that are specifically targeted to use with DNA. All of its modules have counterparts in the RNA menu.   ---   **Help Menu**  The Help menu holds two commands, one to open and browse a detailed help manual, and one to display general information about the RNAstructure application.   ---   **Variable Menus**  Other menus appear on the main menu bar depending on the module selected; these usually appear disabled at first, but are enabled upon successful initialization of a module.   ---   **Quick Access Toolbar**  The toolbar just below the main menu bar holds a group of commonly used commands, including creation of a new sequence and folding of a single strand of RNA. | | |
| Visit The Mathews Lab RNAstructure Page for updates and latest information. | | |
