# Supplementary material for: Dynalign II: common secondary structure prediction for RNA homologs with domain insertions
Source: Nucleic Acids Res. 2014 Nov 21;42(22):13939–48. doi: 10.1093/nar/gku1172 (PMC4267632; doi:10.1093/nar/gku1172)
Supplement: SUPPLEMENTARY DATA [file supp_gku1172_nar-02021-z-2014-File012.zip › manual/GUI/html/Partition_Function.html]

RNAstructure GUI Help -- Partition Function


|  |  |  |
| --- | --- | --- |
|  | RNAstructure GUI Help Partition Function | - Contents - Index |
| The partition function calculation can be used to predict the base pairing probabilities for all possible canonical base pairs in a sequence. The predicted probabilities can be displayed in a probability dot plot, or predicted secondary structures can be color annotated with these probabilities. More probable pairs are more likely to be correctly predicted than less probable pairs.  **Predicting Base Pair Probabilities**   1. Open the partition function window by choosing "RNA -> Partition Function." (A partition function calculation for DNA can be performed with "DNA -> Partition Function DNA.) 2. Choose the sequence by clicking the "Sequence File" button. 3. A save file name will be automatically generated for saving the results. To override this filename, click on the "Save File" button and choose a different filename. 4. Enter any constraints on the partition function calculation from the Force menu, and/or change the calculation temperature, if desired. (See "Tips and Techniques" for details.) 5. Start the calculation by clicking the "Start" button. When the calculation is finished, the probability dot plot window will open. | | |
| Visit The Mathews Lab RNAstructure Page for updates and latest information. | | |
